# User Acceptability and Adoption of AI-Generated Lifestyle Intervention Recommendations: Scoping Review and Theoretical Integration

**DOI:** 10.2196/93573

**Published:** 2026-07-14

**Authors:** Mingjun Ma, Tiange Sui, Shuo Zhou, Lei Shi, Patrick W C Lau

**Affiliations:** 1 Department of Sports and Health Sciences, Academy of Wellness and Human Development, Hong Kong Baptist University, WYS 1130, Dr Wu Yee Sun Building, Baptist University Road CampusChina (Hong Kong), 852 93774078; 2 Department of Social and Behavioural Sciences, City University of Hong Kong, China (Hong Kong); 3 Department of Communication Studies, Hong Kong Baptist University, China (Hong Kong); 4 Laboratory of Exercise Science and Health, Beijing Normal-Hong Kong Baptist University, Zhu Hai, Guang Dong, China

**Keywords:** artificial intelligence, lifestyle interventions, acceptability, adoption, digital health

## Abstract

**Background:**

Artificial intelligence (AI)–generated lifestyle recommendations are increasingly used to support health behavior change. However, AI advice does not necessarily mean that users will accept or adopt those recommendations. Although prior reviews have examined AI-enabled lifestyle interventions and health behavior technologies, fewer have focused on whether users accept and adopt AI-generated recommendations.

**Objective:**

This scoping review aimed to map user acceptability and adoption of AI-generated lifestyle recommendations in user-facing systems used by end users or caregivers. Objectives were to characterize systems and evaluation contexts, clarify how recommendation-level outcomes were conceptualized and measured, synthesize shaping factors, and develop an evidence-informed framework to guide future research, evaluation, and design.

**Methods:**

Following JBI (Joanna Briggs Institute) guidance and the PRISMA-ScR (Preferred Reporting Items for Systematic Reviews and Meta-Analyses extension for Scoping Reviews), we searched Ovid MEDLINE, Ovid Embase, APA PsycInfo via ProQuest, Web of Science Core Collection, Scopus, ACM Digital Library, and IEEE Xplore from database inception to May 5, 2026. The initial search was conducted on November 21, 2025, and an updated search was conducted on May 5, 2026. Eligible studies reported empirical end-user or caregiver data, evaluated AI-generated lifestyle recommendation content delivered without manual review or editing, and reported an acceptability or adoption outcome linked to recommendations. English empirical papers and conference papers were included. Data were charted on study, system, outcome, measurement, factor, and theoretical characteristics. Quality was assessed with the Mixed Methods Appraisal Tool. Findings were synthesized descriptively and through evidence mapping.

**Results:**

Searches yielded 12,997 records; 8570 unique records were screened, and 21 studies were included. Most were published in 2025 or 2026 (17/21, 81%). Large language model–centered systems were the most common format (12/21, 57.1%). Outcomes were concentrated in acceptability-related perceptions, such as satisfaction or enjoyment, perceived quality or fit, and persuasiveness, whereas adoption-related outcomes were assessed less often and mainly reflected intention, in-study uptake, or short-term enactment. Factors clustered across system capabilities, content properties, individual states and capacities, and contextual constraints. Findings informed an integrative perception-intention-enactment framework positioning acceptability and adoption as a system-content-user-context process.

**Conclusions:**

This review extends prior AI and digital health reviews by shifting attention toward how users perceive, intend to follow, and enact AI-generated lifestyle recommendations. Acceptability and adoption appear to depend on systems eliciting and adapting to context, content being actionable and credible, users having the capacity to interpret, trust, and engage with recommendations while retaining control, and resources, routines, and social contexts allowing enactment. The framework can guide theory-driven evaluation, outcome selection, and system design by identifying where recommendation processes may succeed or fail, but should be interpreted as preliminary and evidence-informed rather than causal. By integrating implementation, behavioral, and human-AI perspectives, this review provides a foundation for moving AI-generated lifestyle recommendations from technically plausible outputs toward user-centered, context-sensitive, and behaviorally actionable support.

## Introduction

Noncommunicable diseases (NCDs) are a leading cause of preventable morbidity and mortality worldwide, threatening public health and economies and delaying progress toward the Sustainable Development Goals [1,2]. The burden is increasingly evident earlier in the life course, with substantial and growing deaths and disability among adolescents and young adults [3], and estimates suggest that the unconditional probability of dying from an NCD before age 80 years is 38% for females and 51% for males [1]. Lifestyle-related factors, including physical inactivity, unhealthy dietary patterns, tobacco use, and high alcohol consumption, are key modifiable drivers of NCD risk [4,5], making lifestyle interventions central to prevention and long-term management [6]. Although structured and multidomain interventions improve health behaviors in controlled settings [7], effects often attenuate in routine practice because of barriers to uptake and adherence, implementation constraints, challenges in long-term maintenance, and limited alignment with real-world contexts [6,8].

Digital health technologies have therefore been increasingly adopted to deliver scalable lifestyle and behavior change interventions for NCD prevention and management [9], and advances in artificial intelligence (AI) are accelerating personalized and adaptive lifestyle support [10]. AI-enabled systems include conversational agents and virtual coaches, recommender systems, wearable-based adaptive feedback, and large language model (LLM)–powered conversational systems [10,11], applied across domains such as physical activity (PA), diet and weight management, smoking cessation, and mental health [10]. Evidence suggests that AI-generated lifestyle recommendations can be clinically plausible and reasonably accurate, though with several limitations [12-14], and AI-driven programs may improve health behaviors and related outcomes [10,15].

However, recommendation quality and trial efficacy do not guarantee user acceptability or real-world adoption, particularly when recommendations are generated by AI [16]. Lifestyle change is supported by autonomous motivation and sustained engagement, with self-determination theory-informed evidence linking autonomous motivation to health behavior change [17]. Acceptability and adoption of AI-generated recommendations extend beyond usefulness or feasibility to include trust, transparency, and explainability, autonomy and control, and concerns about privacy, accountability, and ethical risk [18,19]. Importantly, much of the literature emphasizes acceptance of AI systems rather than whether users accept and adopt specific AI-generated recommendations, obscuring how system use translates into recommendation uptake or behavior enactment [20,21]. Accordingly, this review centers on recommendation-level acceptability and adoption and, drawing on an implementation science taxonomy, treats both as implementation outcomes applied to AI-generated recommendation content [22]. In this review, acceptability refers to users’ perceptions and appraisals of AI-generated lifestyle recommendations, whereas adoption refers to users’ intention, decision, uptake, or enactment of the recommended actions [22]. Consistent with the Capability, Opportunity, Motivation–Behavior model (COM-B), these processes may depend on users’ capability to interpret and act on recommendations, motivation to engage with suggested actions, and opportunities or constraints in everyday contexts [23].

User acceptability and adoption of AI-generated lifestyle intervention recommendations are critical, but existing reviews have only partially addressed this issue. Reviews of LLM-based health and exercise coaches have highlighted fragmented evaluation practices and limited real-world user evidence [24], while reviews of diet-related health recommender systems have focused mainly on system functions, recommendation methods, and evaluation criteria, with limited user-centered and longitudinal evaluation [25]. Reviews of AI applications in PA interventions have mapped how AI is used to promote PA, predict PA-related outcomes, and support personalization, monitoring, and adaptation, but have not specifically examined users’ acceptability and adoption of AI-generated advice [11]. Broader reviews of AI acceptance in health care have identified barriers and facilitators across heterogeneous AI technologies, but primarily focus on AI systems rather than on users’ responses to specific AI-generated recommendations [20,21]. Together, these reviews indicate a gap in synthesizing evidence on whether, how, and under what conditions end users or caregivers accept and adopt AI-generated lifestyle recommendations after interacting with user-facing systems.

At the same time, evidence remains fragmented across disciplines and their preferred theoretical lenses, spanning psychological theories (eg, self-efficacy), behavioral science frameworks (eg, the Theoretical Domains Framework), behavior change and persuasive strategies (eg, behavior change techniques; BCT), human-computer interaction (HCI) principles, and computer science work that foregrounds algorithmic performance [18,26-28]. This fragmentation contributes to heterogeneous outcome definitions and measures, limits comparability, and hinders integrative explanations. Moreover, studies often rely on hypothetical scenarios or early-stage design explorations that may not capture acceptability and adoption processes unfolding through real-world use [29]. Given this conceptual and methodological heterogeneity, and the relatively early and rapidly developing evidence base for AI-generated lifestyle recommendation systems, a scoping review is well-suited to mapping how acceptability and adoption have been conceptualized and studied across disciplines [30].

Accordingly, this scoping review focuses on user-facing AI-enabled lifestyle intervention systems used by end users or their caregivers to (1) characterize system formats, AI methods, and evaluation contexts, (2) map how acceptability and adoption outcomes are defined and measured, (3) synthesize factors shaping acceptability and adoption, and (4) integrate identified factors into a preliminary integrative framework to inform future research, system design, and implementation.

## Methods

### Protocol and Registration

A protocol for this scoping review was registered on the OSF (Open Science Framework; bu3ga). The review followed JBI (Joanna Briggs Institute) methodology [31] and the PRISMA-ScR (Preferred Reporting Items for Systematic Reviews and Meta-Analyses extension for Scoping Reviews) guidance [32]. No changes were made to the overall review objectives or Population, Concept, and Context (PCC) framework. During the review process, the protocol concept of AI-generated lifestyle intervention recommendations was further operationalized to improve conceptual clarity and reproducibility. In the synthesis, the protocol categories guided initial coding, while the charted evidence further highlighted individual-level factors and allowed AI-specific issues to be examined across system, content, individual, and contextual levels rather than as a single standalone category. The database searches were updated on May 5, 2026.

### Eligibility Criteria

#### Overview

Eligibility criteria were defined a priori following JBI scoping review guidance and structured using the PCC framework [31].

#### Inclusion Criteria

Studies were eligible if they met all of the following criteria:

1. Population: reported empirical data from human end users (or caregivers).
2. Concept: (1) involved AI-generated recommendation content, including LLM-based systems and systems using other non-LLM AI methods to generate, compose, plan, optimize, adapt, or otherwise construct recommendation content for a specific user rather than merely selecting or ranking items from a predefined library; and (2) AI-generated recommendations had to be delivered to users without manual review or editing of individual recommendations before delivery, such that the AI system controlled recommendation content and/or structure.
3. Context: targeted lifestyle domains, such as PA, diet, sleep, stress management, and smoking cessation.
4. Outcomes: reported at least one acceptability outcome (eg, perception) or adoption outcome (eg, intention or enactment) related to the recommended content. System-use outcomes were eligible only when explicitly interpreted by study authors as reflecting acceptability or uptake of AI-generated recommendations, rather than general engagement with the system.
5. Types of evidence sources: full-text empirical journal papers and conference papers published in English were eligible, with no restrictions on publication year or geographic region.

#### Exclusion Criteria

Studies were excluded if the report was technical-only with no human evaluation; if recommendation content was reviewed, edited, or approved by humans before delivery; if the core logic was rule-based or heuristic, or nonlearning similarity or memory-based; if recommendations were produced solely by selecting or ranking items from a fixed candidate pool (including machine-learning trained rankers); if the context did not involve lifestyle intervention recommendations; or if outcomes were system-level only without a clear link to user acceptability, adoption, or engagement with AI-generated recommendation content. These exclusions were applied because human review or fixed candidate libraries may independently influence recommendation quality, credibility, and fit, thereby complicating interpretation of acceptability and adoption outcomes [25,33].

### Information Sources

#### Overview

Seven electronic databases were searched separately from their earliest available coverage to the latest records available at the time of the final search: Ovid MEDLINE, Ovid Embase, APA PsycInfo via ProQuest, Web of Science Core Collection, Scopus, ACM Digital Library, and IEEE Xplore. Searches were first conducted on November 21, 2025, and updated on May 5, 2026; the most recent search was conducted on May 5, 2026. Database-specific coverage dates and complete search strategies are provided in Multimedia Appendix 1. No registries, supplementary sources, citation searching, contacts, or other search methods were used.

#### Search

Search reporting followed PRISMA-S (Preferred Reporting Items for Systematic Reviews and Meta-Analyses literature search extension) [34]. A PCC-informed search strategy combined terms for (1) AI or generative AI systems; (2) lifestyle or health behavior change domains; and (3) acceptability or adoption outcomes, with additional recommendation or coaching and end-user terms where applicable. Searches combined controlled vocabulary (eg, MeSH [Medical Subject Headings] or Emtree) and free-text keywords in titles or abstracts, using Boolean operators (OR/AND) and database-specific proximity operators as appropriate. Prior syntheses [35] informed the search terms related to AI acceptance and adoption constructs. No database search limits were applied for language, publication year, or publication type. No published search filters were applied, and no formal external peer review of the search strategy was conducted. The updated search was conducted by rerunning the database searches using the same search concepts and database-specific syntax. Complete search strategies as run, along with record counts for all databases, are provided in Multimedia Appendix 1.

### Selection of Sources of Evidence

Records were imported into Rayyan for deduplication and screening [36], with duplicate records removed before screening. Study selection followed a two-stage process (title or abstract screening, then full-text review) using the prespecified eligibility criteria. MM and TS independently screened all records at both stages. Discrepancies were resolved through discussion until consensus was reached, with consultation of a third reviewer (PWCL) when needed. For reports excluded at the full-text stage, the primary reason for exclusion was documented. The study selection process is reported in the PRISMA (Preferred Reporting Items for Systematic Reviews and Meta-Analyses) flow diagram, and a list of full-text exclusions with reasons is provided in Multimedia Appendix 1. Substantively distinct reports were retained as separate records and charted independently when evaluations differed in focus, outcomes, or study procedures.

### Data Charting Process

Data were charted in Microsoft Excel using a standardized form developed a priori and refined during piloting. MM charted all studies, and TS independently verified all fields and coding decisions for accuracy and completeness. Disagreements were resolved through discussion until consensus was reached, with PWCL adjudicating when needed. No study investigators were contacted to obtain or confirm data.

### Data Items

Data items included study characteristics (authors, publication year, country or region, publication type, study design, sample size, participant characteristics, and lifestyle domain); system characteristics (system format, core AI method, data inputs, knowledge sources, and delivery channel); recommendation-level outcome constructs, including acceptability-related perceptions, adoption-related intentions, behavioral choice or enactment, and formative or contextual evidence; measurement or evidence approaches; reported factors shaping acceptability and adoption; and explicit theories, models, frameworks, or strategy lenses used in design, evaluation, or interpretation.

### Critical Appraisal of Individual Sources of Evidence

The methodological quality of the included studies was independently appraised by MM and TS using the Mixed Methods Appraisal Tool (MMAT) [37]. The MMAT applies five core methodological criteria appropriate to the study design (eg, qualitative, quantitative, or mixed methods). Each of the five design-appropriate MMAT criteria was rated (yes, no, or cannot tell). For descriptive reporting, the number of criteria met was summarized as a 0‐5 score (with the corresponding percentage), consistent with the 2020 guidance. Discrepancies were resolved through discussion, with PWCL adjudicating if needed. Item-level MMAT assessments are provided in Multimedia Appendix 2 [18,19,26-28,38-53].

### Synthesis of Results

Charted data were summarized descriptively using tables, figures, frequencies, and percentages where appropriate. Following guidance for analysis and presentation of results in scoping reviews, factors shaping acceptability and adoption were synthesized using a deductive-inductive mapping approach: protocol-informed categories guided initial coding, while additional factor groups were refined through repeated comparison of the charted evidence [54]. Recommendation-level outcomes were mapped to acceptability, adoption, and formative or contextual evidence categories. Evidence maps were generated to visualize outcome constructs and factor groups by measurement or evidence type. Descriptive summaries and visualizations were produced using R (version 4.4.2; R Foundation).

## Results

### Study Selection

**Figure 1.**
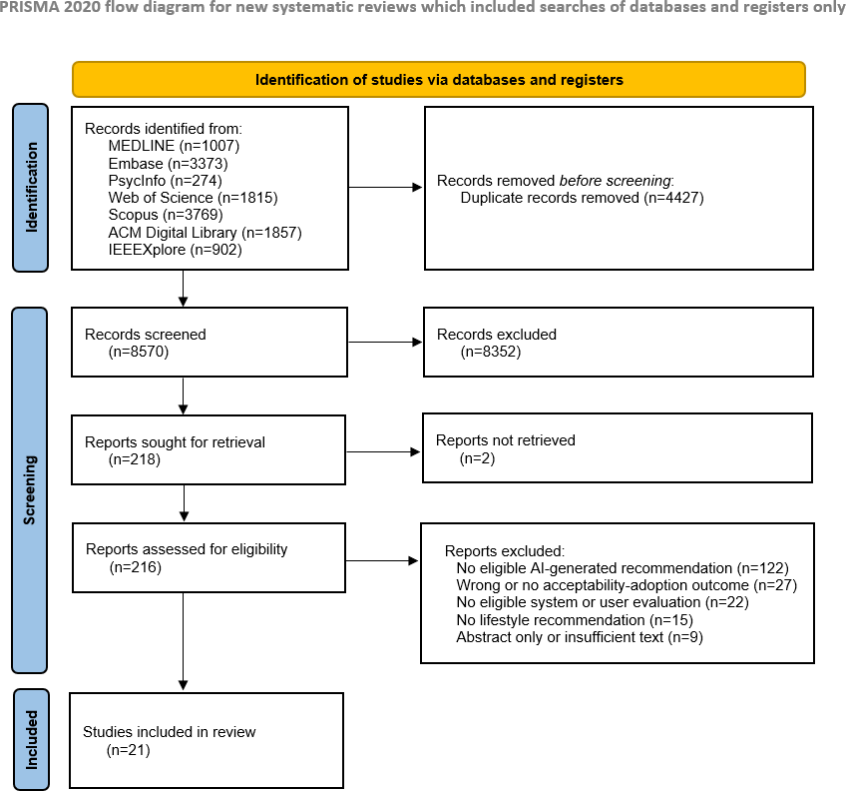
PRISMA flow diagram of study selection. The diagram shows the identification, screening, eligibility assessment, and inclusion of studies on AI-generated lifestyle recommendations. AI: artificial intelligence; PRISMA: Preferred Reporting Items for Systematic Reviews and Meta-Analyses.

The database searches yielded 12,997 records. After removal of duplicates, 8570 unique records were screened. The large duplicate count likely reflected overlap across multidisciplinary databases. Title and abstract screening excluded 8352 records, and 218 reports were sought for retrieval. Two reports could not be retrieved; therefore, 216 full-text reports were assessed for eligibility. A total of 195 reports were excluded at full-text review. The most common exclusion reason was no eligible AI-generated recommendation, mainly referring to rule-based, heuristic, similarity-based, or fixed-candidate selection or ranking systems rather than recommendations constructed by AI for individual users. Other reasons included irrelevant or absent acceptability or adoption-related outcomes, no eligible system or user evaluation, no lifestyle recommendation, and abstract-only or insufficient full-text availability. Finally, 21 studies met the inclusion criteria and were included in the synthesis (Figure 1).

### Characteristics of Included Studies

Included reports were published between 2022 and 2026, with the evidence base concentrated in 2025 or 2026 (17/21). Six reports were journal papers [19,38-42], and 15 reports were conference papers [18,26-28,43-53]. Studies were conducted across 12 countries or regions, including the United States (n=3) [18,42,47], and Canada [19,27], mainland China [28,52], Germany [43,46], Hong Kong, China [26,45], India [49,53], Ireland [39,48], and Singapore [41,50] (n=2 each), and Japan [51], Norway [44], the United Kingdom [38], and Vietnam [40] (n=1 each).

Study designs included quantitative descriptive studies (n=5) [28,38,40,41,53], mixed-methods studies (n=5) [18,19,27,50,52], qualitative studies (n=4) [43,44,46,47], nonrandomized studies (n=4) [42,45,49,51], and randomized trials (n=3) [26,39,48]. Across lifestyle domains, diet-related recommendations were most common, including nutrition-focused dietary guidance (n=6) [26,28,40,45,51,52] and weight-loss-focused dietary support (n=2) [19,27]. Further, 4 studies examined combined diet and PA recommendations [38,41,46,53], 3 studies targeted PA recommendations alone [39,44,48], 3 studies addressed multidomain lifestyles [43,49,50], and single studies addressed PA-sleep [18], stress management [47], and sleep [42].

All studies evaluated end users, and sample sizes ranged from 2 [47] to 366 [43] participants. Methodological quality varied across studies, with 15 studies meeting four or five MMAT criteria [18,19,26,27,38-41,43-48,51] and 6 studies meeting one to three criteria [28,42,49,50,52,53] (Table 1).

**Table 1.** Overview of included studies and extracted characteristics. This table summarizes the study design, country or region, publication type, sample size, participant characteristics, and lifestyle domain for each included study. Included studies were published from 2022 to 2026 and examined AI^a^-generated lifestyle recommendations across diet, PA^b^, sleep, stress management, weight management, smoking cessation, or multidomain lifestyle contexts.

Study	Country	Publication type	Study design	Value, n^c^	Participants	Lifestyle domain	MMAT^d^^,^^f^
Alcaraz-Herrera et al, 2022 [38]	UK	Journal paper	Descriptive	205; subsequent 44	14‐49 y, both sexes	Diet and PA	5^e^
Ataguba and Orji, 2025 [19]	Canada	Journal paper	Mixed	17	≥18 y, 11M^g^/6F^h^, ChatGPT users, weight-loss history	Diet/weight loss	5^e^
Ataguba et al, 2025 [27]	Canada	Conference paper	Mixed	17	≥18 y, weight-loss plans, ChatGPT experience	Diet/weight loss	5^e^
R et al, 2026 [49]	India	Conference paper	Nonrandomized	NR^i^	Patients with Parkinson disease, mild to advanced stages	Multidomain	2^j^
Doherty et al, 2024 [39]	Ireland	Journal paper	RCT^k^	62	18‐65 y, 24M/38F, healthy active adults	PA	5^e^
Gao et al, 2026 [50]	Singapore	Conference paper	Mixed	8	68‐86 y, 5F/3M, older adults living alone	Multidomain	3^l^
Gao et al, 2025 [51]	Japan	Conference paper	Nonrandomized	12	23‐32 y, 4F/8M	Diet/nutrition	5^e^
Goh et al, 2026 [41]	Singapore	Journal paper	Descriptive	20	40‐59 y, 10F/10M, community residents	Diet and PA	5^e^
Jahn et al, 2025 [43]	Germany	Conference paper	Qualitative	366	≥65 y	Multidomain	5^e^
Larbi et al, 2025 [44]	Norway	Conference paper	Qualitative	5	20‐60 y, chatbot experience	PA	5^e^
Liang et al, 2025 [26]	Hong Kong, China	Conference paper	RCT	214 (follow-up 140)	18‐66 y, 129F/84M, interested in diet	Diet/nutrition	4^m^
Liang and Luo, 2025 [45]	Hong Kong, China	Conference paper	Nonrandomized	161	18‐69 y, 84F/77M, dietary preferences/restrictions	Diet/nutrition	4^m^
Liu et al, 2025 [28]	China	Conference paper	Descriptive	50	NR	Diet/nutrition	2^j^
Liu and Liu, 2026 [42]	US	Journal paper	Nonrandomized	42	18‐75 y, 12M/30F, poor sleep	Sleep	3^l^
Meywirth, 2024 [46]	Germany	Conference paper	Qualitative	11	NR	Diet & PA	5^e^
Neupane et al, 2025 [47]	US	Conference paper	Qualitative	2	PhD students, high stress	Stress management	5^e^
Phan et al, 2026 [40]	Vietnam	Journal paper	Descriptive	265	18‐39 y, 190F, most had ChatGPT experience	Diet/nutrition	5^e^
Sheng et al, 2025 [52]	China	Conference paper	Mixed	186	NR	Diet/nutrition	2^j^
Tragos et al, 2023 [48]	Ireland	Conference paper	RCT	69	Adults, 27M/42F, recreational exercisers	PA	4^m^
Tyagi et al, 2025 [53]	India	Conference paper	Descriptive	10	20‐30 y, beta users	Diet and PA	1^n^
Wang et al, 2025 [18]	US	Conference paper	Mixed	16	18‐44 y, 10M/5F, motivated to improve sleep/PA	PA/sleep	4^m^

a AI: artificial intelligence.

b PA: physical activity.

c For studies with multiple sample-size entries, “subsequent” and “follow-up” indicate participants who completed an additional challenge study or follow-up survey after the main evaluation.

d MMAT: Mixed Methods Appraisal Tool.

e 5: each count represents 20% of the quality appraisal standards met.

f Mixed Methods Appraisal Tool values indicate the number of applicable criteria met by each study, with a maximum of 5; higher values indicate that more criteria were met.

g M: male.

h F: female.

i NR: not reported.

j 2: each count represents 20% of the quality appraisal standards met.

k RCT: randomized controlled trial, including randomized crossover trials.

l 3: each count represents 20% of the quality appraisal standards met.

m 4: each count represents 20% of the quality appraisal standards met.

n 1: each count represents 20% of the quality appraisal standards met.

### System Characteristics and Design Features

**Figure 2.**
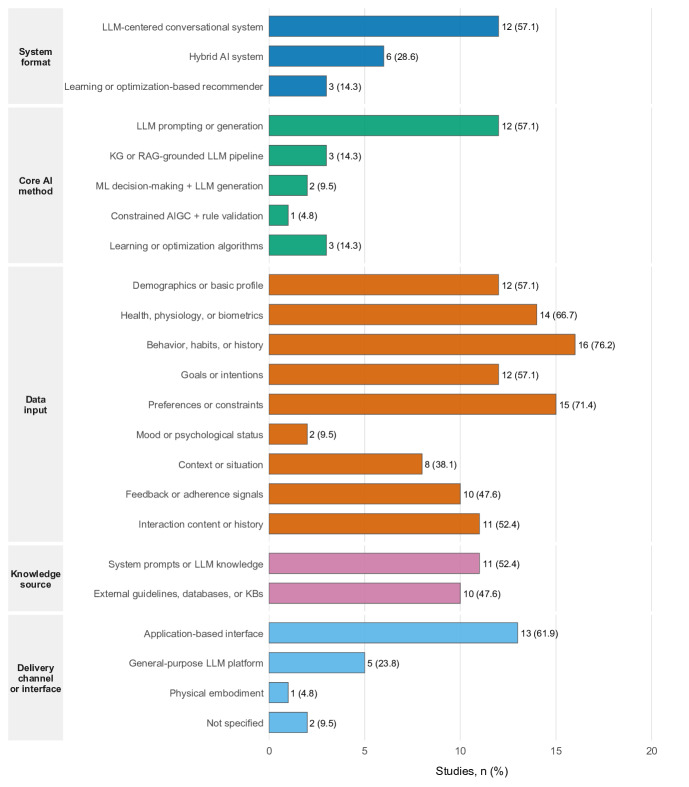
Distribution of system characteristics and feature inputs across included studies. Bars indicate the number of studies in each category, with percentages calculated using the total number of included studies as the denominator (n=21). For system format and core AI method, each study was assigned to the most representative category. Data input categories were not mutually exclusive because a single system could use multiple types of user information. AI: artificial intelligence; AIGC: artificial intelligence–generated content; KG: knowledge graph; LLM: large language model; ML: machine learning; RAG: retrieval-augmented generation.

The included systems varied in format, AI method, input data, knowledge grounding, and delivery channel (Figure 2). Study-level system characteristics are provided in Multimedia Appendix 1. LLM-centered conversational systems were the most common system format (12/21, 57.1%), followed by hybrid AI systems (6/21, 28.6%) and learning or optimization-based recommenders (3/21, 14.3%). At the method level, LLM prompting or generation was the most frequently reported core AI method (12/21, 57.1%). Smaller subsets of studies implemented knowledge graph– or retrieval-augmented generation–grounded LLM pipelines (3/21, 14.3%), learning or optimization algorithms (3/21, 14.3%), machine learning (ML)–supported decision-making combined with LLM generation (2/21, 9.5%), or constrained artificial intelligence–generated content with rule validation (1/21, 4.8%).

Personalization most often relied on user behaviors and habits (16/21, 76.2%), preferences and constraints (15/21, 71.4%), and health, physiology, or biometrics (14/21, 66.7%). Demographics or basic profiles and goals or intentions were each used in 12 (57.1%) studies. Interaction content or history was used in 11 (52.4%) studies, feedback or adherence signals in 10 (47.6%) studies, context or situation in 8 (38.1%) studies, and mood or psychological status in 2 (9.5%) studies. Knowledge sources were nearly evenly split between system prompts or LLM knowledge (11/21, 52.4%) and external guidelines, databases, or knowledge bases (10/21, 47.6%). Delivery most often occurred via application-based interfaces (13/21, 61.9%), followed by general-purpose LLM platforms (5/21, 23.8%), with physical embodiment and unspecified delivery channels reported less frequently.

In summary, the evidence base was characterized by LLM-centered conversational systems that drew on extensive user profiling, especially behavioral histories, preferences, constraints, and health-related information. However, systems varied in how recommendations were grounded, optimized, adapted, and delivered.

### Acceptability and Adoption Constructs and Measurement Approaches

**Figure 3.**
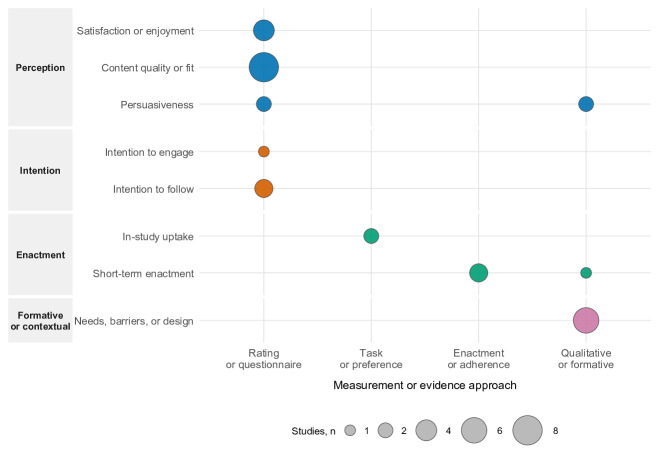
Evidence map of recommendation-level outcomes by measurement approach. Rows represent outcome construct groups, and columns represent the measurement or evidence approach used in the included studies. Bubble size indicates the number of studies contributing evidence to each outcome construct–measurement approach cell. Empty or small cells indicate areas where evidence was limited.

Across included studies, recommendation-level outcomes were organized as acceptability, adoption, and formative or contextual evidence (Table 2). Acceptability was mainly reflected in perceptual appraisals of recommendation content, while adoption was reflected in intention, in-study uptake, and short-term enactment of recommended actions. The evidence base emphasized acceptability-related perceptual outcomes and intention-related measures, whereas enactment-related indicators were less frequently assessed. Figure 3 visualizes the distribution of these outcome constructs by measurement or evidence approach.

**Table 2.** Conceptual mapping of recommendation-level outcomes to acceptability and adoption. Definitions of acceptability and adoption were adapted from the implementation outcomes taxonomy by Proctor et al [22] and applied at the level of AI^a^-generated lifestyle recommendations. Overlapping appraisals of recommendation content were grouped as acceptability-related indicators, consistent with previous discussion of overlap between acceptability and appropriateness [22].

Implementation outcome/evidence type	Definition applied in this review	Outcome manifestation in included studies	Outcome construct groups
Acceptability	Users’ perception and appraisal of AI-generated lifestyle recommendations, including whether recommendations were satisfactory, relevant, fitting, persuasive, useful, or appropriate.	Perception	Satisfaction/enjoyment [28,38,39,48]; content quality/fit [26,38,41,42,45,51-53]; persuasiveness [19,27]
Adoption	Users’ intention, decision, or action to try, use, follow, or implement AI-generated lifestyle recommendations.	Intention	Intention to engage [40]; intention to follow [26,45,50]
Adoption	Users’ intention, decision, or action to try, use, follow, or implement AI-generated lifestyle recommendations.	Enactment	In-study uptake [38,45]; short-term enactment [18,26,49,50]
Formative/contextual evidence	Evidence explaining user needs, barriers, design preferences, and reasons related to acceptability and adoption.	Explanatory or contextual evidence	Needs/barriers/design principles [43,44,46,47,50,52]

a AI: artificial intelligence.

### Perception

Perceptual and attitudinal outcomes formed the largest body of evidence. Satisfaction or enjoyment was the most frequently assessed construct and was measured using both standardized instruments and custom measures. In the PA domain, enjoyment was assessed using the Physical Activity Enjoyment Scale (8-item version) [39,48]. Studies focusing on dietary or mixed lifestyle recommendations relied primarily on researcher-developed Likert-type ratings targeting specific recommended content or interaction sessions [28,38].

Perceived content quality or fit was another commonly assessed perceptual construct. This dimension was operationalized either through custom ratings of content attributes, such as diversity, healthiness, relevance, or attractiveness [38], or through adapted and study-developed questionnaires assessing perceived recommendation accuracy, quality, novelty, diversity, explanation quality, expectation fit, personalization, appropriateness, actionability, tailoring, rationale understanding, and goal or input fit [26,41,42,45,51-53]. In addition, some studies examined perceived persuasiveness, defined as the extent to which AI-generated recommendations were perceived as capable of influencing users’ attitudes or behaviors. This construct was assessed using adapted versions of the Perceived Persuasiveness Scale and was often accompanied by open-ended questions or interviews to contextualize variation in persuasiveness ratings [19,27].

### Intention

Intention was examined at two levels. Intention to engage (to receive recommendations) captured users’ willingness to continue using a system to receive AI-generated recommendations and was measured using adapted intention-to-engage scales [40]. Intention to follow recommendations reflected users’ willingness to take up the recommended actions and was operationalized through contextualized, researcher-developed questionnaire items referring explicitly to the recommended content [26,45,50].

### Enactment

Only a small number of studies assessed behavioral enactment. In-study uptake was measured through immediate indicators such as recommendation acceptance rates [45] or blind paired forced-choice preference tasks comparing alternative recommendation systems [38]. Short-term enactment was evaluated via brief follow-up measures, including retrospective categorical uptake, binary daily adherence items, and patient self-reports with caregiver verification [18,26,49]. Qualitative uptake evidence further indicated whether recommended diet or exercise actions were implemented or integrated into daily routines during longitudinal interaction with the system [50]. As shown in Figure 3, enactment-related evidence was less common than perceptual and intention-related evidence.

### Formative or Contextual Evidence

In addition to outcome-focused measurements, several qualitative studies contributed formative and exploratory evidence. Drawing on empirical interaction with system prototypes or functional components, these studies used semistructured interviews, focus groups, think-aloud protocols, prototype evaluations, auto- or duoethnographic methods, open-ended responses, and short interviews to examine how AI-generated lifestyle recommendations were appraised, trusted, followed, or not taken up [43,44,46,47,50,52]. Rather than operationalizing acceptability or adoption as discrete outcomes, this body of work focused on identifying user needs, contextual barriers, cultural considerations, and design principles relevant to acceptability and adoption.

Taken together, Figure 3 shows that outcome evidence was concentrated in perceptual appraisal of recommendation content, especially satisfaction or enjoyment, and content quality or fit. Adoption-related evidence was less common and was distributed across intention, in-study uptake, and short-term enactment. Qualitative or formative evidence provided important explanatory context but was less often linked to discrete acceptability or adoption outcome measures. This pattern suggests that existing studies more often assessed whether users positively appraised AI-generated lifestyle recommendations than whether users went on to try, follow, or take them up.

### Factors Shaping Acceptability and Adoption

**Figure 4.**
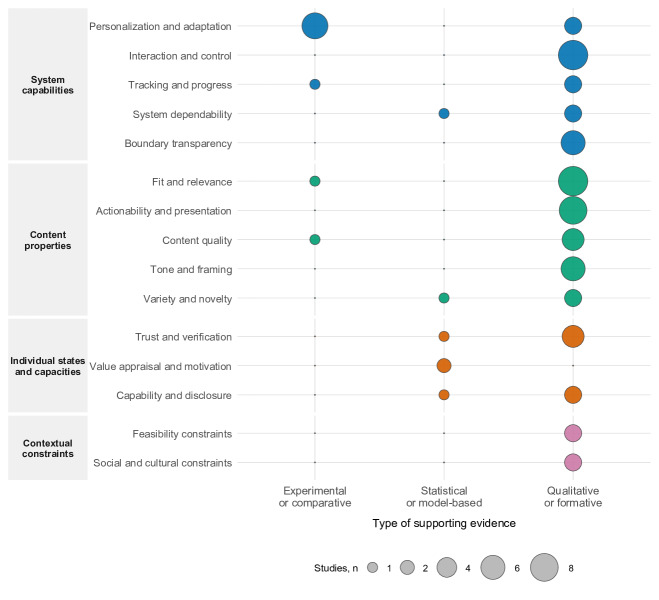
Evidence map of factor groups by supporting evidence type. Rows represent factor groups synthesized from included studies, and columns represent the type of supporting evidence reported in the included studies. Bubble size indicates the number of studies contributing evidence to each factor group–evidence type cell. The map shows the distribution of evidence supporting the factor map and does not imply confirmed causal predictors or stage-specific effects.

Across studies, reported factors shaping acceptability and adoption spanned system capabilities, content properties, individual states and capacities, and contextual constraints, including 13 core factors (Table 3). Quantitative tests of moderation or mediation were uncommon, and factors were most often reported as experimental or comparative manipulations, quantitative associations, or qualitative or formative themes (Figure 4). Accordingly, these factors were interpreted as evidence-informed conditions in the recommendation process rather than confirmed causal predictors or stage-specific effects.

**Table 3.** Multilevel factor map shaping the acceptability and adoption of artificial intelligence–generated lifestyle recommendations. Factors are interpreted as evidence-informed conditions in the recommendation process, rather than as confirmed causal predictors or stage-specific effects.

Level and core factor	Synthesized role
System capabilities	
A1. Personalization and adaptation	Dynamic tailoring and iterative refinement based on user goals, preferences, abilities, profiles, feedback, interaction histories, constraints, and context. This includes optimization, adaptive sequencing, alternative generation, and knowledge-supported personalization [18,19,28,38,39,46-48,51,52].
A2. Interaction and control	Elicitation, clarification, low-burden input, refinement options, customization, override controls, frequency preferences, and optional or cautious modes that allow users to shape recommendations and interaction style [18,27,41,43,44,46,47,50,52].
A3. System infrastructure and dependability	
A3a. Tracking/progress features	Built-in tracking, reminders, progress summaries, rewards, monitoring feedback, and follow-up mechanisms that support self-monitoring and follow-through [18,27,43,49].
A3b. System dependability	Technical stability, reliable data access, platform credibility, and accurate measurement or sensing that support dependable system use [18,40,43,44].
A3c. Boundary-condition transparency	Transparency about privacy, data use, system assumptions, recommendation generation, AI involvement, and system constraints or limitations [18,41,43,44,47,52].
Content properties	
B1. Fit/relevance	Alignment of recommendations with users’ goals, preferences, constraints, habits, cultural context, health needs, domain needs, and daily routines [18,19,27,41,44,46,47,49,50,52].
B2. Actionability and presentation	Concrete, structured, specific, concise, and multimodal guidance, including implementation details, visual aids, and formats that make recommendations easier to understand and act on [18,19,27,41,44,46,47,50].
B3. Content quality: coherence, explainability, and correctness	Coherence, consistency, correctness, safety, explainability, rationale, evidence cues, domain accuracy, and avoidance of contradictory or unsupported advice [18,19,27,28,46,52].
B4. Tone and framing	Autonomy-supportive, motivational, emotionally supportive, nondirective, and appropriately warm or authoritative framing, including suggestion-based rather than command-based communication [18,27,43,46,47,50].
B5. Variety/novelty	Diversity, novelty, and avoidance of repetitive recommendation content to reduce fatigue and sustain interest over repeated use [18,19,47,48].
Individual states and capacities	
C1. Trust and verification	Conditional trust, credibility judgments, privacy concerns, authenticity concerns, expert-review expectations, and users’ need to verify AI-generated recommendations before relying on them [19,27,40,47,50,52].
C2. Value appraisal and motivation	Cost-benefit value appraisal, motivational readiness, habitual routines, and willingness to try or follow suggested actions [26,40].
C3. Capability and disclosure	Prompt literacy, technological literacy, communication ability, accessibility needs, and willingness to disclose personal or sensitive context needed for tailored recommendations [19,26,27,50].
Contextual constraints	
D1. Feasibility constraints	Practical limits related to time, cost, resources, availability, facilities, location, accessibility, health status, travel, illness, deadlines, and other life circumstances [18,26,27].
D2. Social and cultural constraints	Family responsibilities, decision agency, social endorsement, cultural values, and social or cultural expectations shaping whether recommendations fit user’s lived context [19,26,43].

### System Capabilities

Acceptability and adoption were more consistently supported when systems enabled dynamic updating or iterative refinement of recommendations based on feedback, goals, abilities, user profiles, preferences, interaction histories, constraints, and context, rather than static outputs or insufficiently tailored recommendations [18,19,28,38,39,46-48,51,52]. In contrast, one study suggested no statistically significant relationship between personalization and acceptance [40].

Interaction design and user control, such as elicitation or clarification flows, low-burden input, customization, refinement options, style or tone adjustability, user say in plan refinement, configurable or cautious automation modes, and override or frequency controls, were repeatedly framed as reducing friction and supporting acceptability and adoption [18,27,41,43,44,46,47,50,52], whereas limited refinement, insufficient follow-up support, and inadequate context elicitation were discussed as barriers [18,44,46,47].

Tracking or progress features, including summaries, rewards, reminders, monitoring feedback, and wearable-based feedback loops, were described as facilitating acceptability and adoption [18,27,43,49]. Finally, system dependability (stability, data access, platform credibility, and measurement accuracy) and constraint or privacy transparency were treated as prerequisites for reliable reliance, shaping whether users could appropriately interpret outputs and decide what to act on [18,40,41,43,44,52]. Separately, a system-architecture comparison (multi-LLM vs single-LLM) reported no significant effect [45].

As shown in Figure 4, system-level evidence included the strongest experimental or comparative support for personalization and adaptation, whereas interaction or control, boundary transparency, tracking or progress, and dependability were supported mainly by qualitative or formative evidence.

### Content Properties

Across studies, evaluations centered on whether outputs achieved fit and relevance to goals, preferences, constraints, cultural context, and domain needs, where mismatches were described as impairing acceptability and adoption [19,27,41,44,46,47,49,50,52].

Actionability and presentation, including concrete steps, structured formats, multimodal aids, specific feedback, visual demonstrations, and detailed implementation guidance, were repeatedly linked to positive perception and enactment likelihood, while text-only or underspecified guidance weakened acceptability and adoption [18,19,27,41,44,46,47,50].

For content quality, coherence or interpretability, and justification cues emerged as salient factors. Specifically, ablation evidence showed that removing structured prompting or constraint components reduced coherence or interpretability and satisfaction [28], and qualitative accounts reported lower confidence when outputs were inconsistent, inaccurate, lacked source or verification cues, showed unclear goal understanding, provided insufficient rationale, or raised safety-related concerns [18,19,27,46,52].

Tone or framing also mattered, with autonomy-supportive, nonimposing preferred, emotionally supportive, suggestion-based, teacher-like, copilot, or assistant framing described as most acceptable when paired with actionable guidance [18,27,43,46,47,50]. Variety or novelty was linked to acceptability, while repetitiveness and novelty decay were discussed as fatigue-related barriers [18,19,47,48].

Figure 4 shows that content-level factors were supported predominantly by qualitative or formative evidence across fit or relevance, actionability or presentation, content quality, tone or framing, and variety or novelty, with limited experimental or comparative, or statistical or model-based evidence for selected content factors.

### Individual States and Capacities

Trust-related factors emerged as proximal factors, including conditional trust and the need for verification, and privacy-related uncertainty [19,47,52]. Notably, greater technological literacy did not necessarily translate into uncritical reliance; technologically confident users could remain skeptical and prefer to independently verify AI-generated recommendations before acting on them [50]. In addition, one study empirically tested a mediation pathway from perceived value to skepticism to acceptance [40]. Value appraisal and motivational or habitual readiness were treated as near-term drivers of adoption. Intention to follow recommendations showed a positive association with adoption, while low motivation or habits constrained follow-through [26,40]. Prompt literacy barriers and willingness to disclose sensitive information were described as shaping whether sufficient usable context was provided during interaction, with downstream implications for acceptability and adoption [19,26,27]. Structured guidance could help users with lower technological literacy express needs and engage with recommendations more confidently [50].

As shown in Figure 4, individual-level factors included more statistical or model-based evidence than other levels, particularly for trust or verification, value appraisal or motivation, and capability or disclosure, although qualitative or formative evidence also contributed to trust and disclosure-related factors.

### Contextual Constraints

Enactment was consistently bounded by feasibility constraints (resources, time, facilities, and life events), which limited uptake even when recommendations were perceived as relevant [18,26,27]. Social and cultural constraints (family responsibility, decision agency, cultural values, and endorsement cues) further shaped the real-world conditions under which recommendations could be adopted [19,26,43].

Figure 4 shows that contextual constraints were supported mainly by qualitative or formative evidence, indicating that these factors were typically identified through users’ accounts of feasibility, social, and cultural barriers rather than through quantitative testing.

In summary, Figure 4 shows that qualitative or formative evidence supported most factor groups, whereas experimental or comparative and statistical or model-based evidence were concentrated in fewer areas. This distribution supports interpreting the factor map as a synthesis of evidence-informed conditions shaping recommendation-level acceptability and adoption, rather than as confirmed causal determinants.

### Theoretical Grounding and Framework Used Across Studies

Across the included studies, explicit theoretical or strategy lenses were used in a minority of cases and typically served to structure interpretation, content coding, or design derivation rather than to test a full causal theory of adoption. Two studies articulated psychological mechanism accounts linking intermediate perceptions to acceptability-related intentions: Phan et al [40] drew on Integrated Fear Acquisition Theory to position consumer skepticism as an affectively grounded resistance mechanism, modeling how perceived platform credibility and perceived value reduce skepticism and thereby increase intention to engage, while perceived personalization or relevance showed nonsignificant effects. Liang et al [26] applied the Bandura self-efficacy perspective post hoc to interpret the observed association between intention to follow recommendations and subsequent adherence.

Five studies applied strategy-oriented lenses to characterize recommendation content and derive actionable implications: Ataguba and Orji [19] and Ataguba et al [27] organized formative evaluation around persuasive-strategy features (discussed via Persuasive Systems Design strategies), Wang et al [18] used the Theoretical Domains Framework to select chatbot-deliverable BCTs and assess strategy coverage in generated interactions, and Meywirth [46] explicitly used Persuasive Systems Design to derive autonomy-supportive, nonimposing recommendation principles. Gao et al [50] used a theory-informed persona-based persuasive design approach, combining health behavior change, personality, empathy-based design, and persuasion-matching principles to guide culturally sensitive persona construction and recommendation framing. The remaining studies did not report an explicit theory or framework and instead relied on study-specific assumptions, formative user needs work, and domain knowledge from prior literature.

## Discussion

### Principal Findings

**Figure 5.**
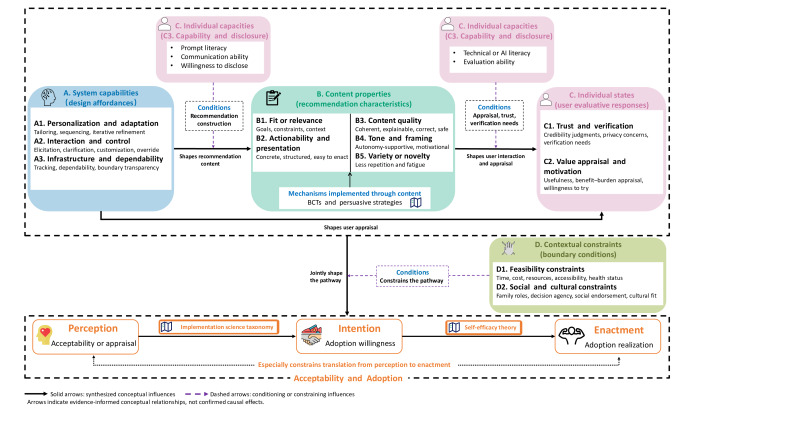
Integrative framework of acceptability and adoption of AI-generated lifestyle intervention recommendations. The framework was synthesized from the outcome mapping and multilevel factor synthesis of included studies. System capabilities, content properties, and individual evaluative states are shown as upstream components that jointly shape the acceptability and adoption pathway. Capability and disclosure are displayed as pathway-conditioning factors: input capability and disclosure condition recommendation construction, whereas technology literacy and verification orientation condition appraisal, trust, and verification. Contextual constraints are shown as boundary conditions that may constrain the pathway as a whole, especially the translation from intention to enactment. BCTs and persuasive strategies are positioned within content properties because they are delivered through recommendation content. Solid arrows indicate synthesized conceptual influences; dashed arrows indicate conditioning or constraining influences. Arrows represent evidence-informed conceptual relationships and do not imply confirmed causal effects. AI: artificial intelligence; BCT: behavior change technique.

This scoping review synthesized evidence from 21 studies examining the acceptability and adoption of AI-generated lifestyle intervention recommendations in user-facing systems used by end users. The included studies varied in system format, core AI method, data inputs, knowledge source, and delivery channel or interface across lifestyle domains, but most evidence remained concentrated on short-term, formative, or self-reported evaluations. Acceptability was primarily represented by users’ perceptions or appraisals of the recommended content, whereas adoption was represented by intention, in-study uptake, or short-term enactment, with limited evidence on sustained enactment. Factors shaping acceptability and adoption clustered across system capabilities, content properties, individual states and capacities, and contextual constraints. These findings informed the integrative framework in Figure 5, which organizes the mapped evidence as a perception-intention-enactment pathway shaped by upstream system, content, user, and contextual conditions.

### Interpretation and Comparison With Prior Work

#### Interpretation of the Integrative Framework

Figure 5 should be read as an evidence-informed conceptual synthesis rather than a tested causal model [54]. Whereas broader health care AI acceptance reviews have synthesized factors associated with acceptance or use of AI systems and technologies [20,21], this framework shifts attention to the specific AI-generated recommendation content that users appraise, intend to follow, or enact. The framework suggests that system capabilities, content properties, and individual evaluative states jointly shape the perception-intention-enactment pathway, while also influencing one another [26,46,50]. System capabilities may shape recommendation content through personalization, adaptation, elicitation, and iterative refinement, and may shape user states through interaction design, user control, dependability, and transparent delivery [19,43,51]. Content properties may then shape user states and appraisal through fit, actionability, explainability, framing, and novelty [19,46,52]. In this structure, BCTs and persuasive strategies are positioned within content properties because they are delivered through the recommendation content, rather than treated as a separate factor domain [18,27,50].

Capability and disclosure are shown as pathway-conditioning factors because they influence the translation between layers, not merely users’ postexposure evaluations [26,27,40,50]. Input capability and disclosure condition whether system capabilities can become personalized recommendation content by shaping how users communicate goals, preferences, constraints, and sensitive context [19,26,27]. Technology literacy and verification orientation condition how recommendation content is interpreted, questioned, trusted, or verified [40,50,52]. Contextual constraints are positioned as boundary conditions because they may shape how system capabilities, content properties, and individual evaluative states translate into acceptability and adoption outcomes and may also constrain transitions within the outcome pathway, especially from intention to enactment [18,26,43]. Therefore, enactment in this framework indicates recommendation adoption only when linked to AI-generated recommendation content, not general intervention effectiveness [22].

#### Interpreting the Findings Through Existing Theories and Research

Existing technology acceptance theories help explain why system capabilities emerged as important in this review. The Technology Acceptance Model emphasizes perceived usefulness and perceived ease of use as determinants of information technology acceptance [55], while the Unified Theory of Acceptance and Use of Technology also identifies expected performance and effort as important factors shaping technology use [56]. These constructs help interpret system-level findings in this review, including personalization, low-burden interaction, user control, dependability, and transparent delivery [19,43,51]. However, AI-generated lifestyle recommendations require users to appraise not only whether a system is useful or usable, but also whether specific generated recommendations are relevant, actionable, explainable, appropriately framed, and sufficiently varied to support continued engagement [19,46,52]. This distinction explains why content properties were integrated into the recommendation process, because continued system use does not necessarily mean that users accept, trust, or enact the specific AI-generated recommendations they receive [26,40,50].

HCI and human-AI interaction perspectives further help explain the interaction and content layers of the framework. Human-AI interaction guidelines emphasize setting appropriate expectations, supporting efficient interaction, enabling user control, and helping users understand system capabilities and limitations [57]. These concerns align with the mapped importance of interaction burden, refinement options, transparency, explanation quality, and boundary-condition communication [18,43,46]. Prior work on AI literacy also supports treating capability and disclosure as pathway-conditioning factors, because AI literacy involves competencies for understanding, critically evaluating, and effectively interacting with AI systems [58]. In this review, these capacities appeared in applied forms, including input capability, prompt or technology literacy, willingness to disclose context, and verification orientation [19,26,27,50].

Finally, behavior change theory helps explain why recommendation-level acceptability and adoption should be understood as a system-content-user-context process rather than as an attitude toward AI alone. The COM-B model explains behavior (B) as arising from the interaction of capability (C), opportunity (O), and motivation (M) [23], while suggesting AI-specific extensions. In this evidence base, capability extends beyond lifestyle-related skills to include interaction-related abilities, such as prompt literacy and providing information that enables meaningful personalization [19,26,27,50]. Opportunity is shaped not only by external environments but also by the interaction environment created by AI systems, including interaction burden, adaptive updating, timing, and availability of alternatives under constraints [18,43,46,51]. Motivation incorporates AI-specific processes such as trust calibration, perceived autonomy support, and engagement dynamics related to novelty, fatigue, and perceived control [19,46,50,52]. Together, these observations suggest that acceptability and adoption depend on how users interact with and interpret AI systems over time and how system-mediated conditions expand or constrain feasible action.

#### Implications for System Design and User Evaluation Research

The framework suggests that AI-generated lifestyle recommendation systems should support the whole recommendation process, rather than only produce plausible advice. At the system level, guided elicitation, low-burden input, refinement options, user control, and transparent boundary cues may help users provide information needed for personalization while maintaining autonomy and calibrated trust [18,43,46]. Such guidance may be particularly important for users with lower technological literacy, for whom structured interaction can support need expression and more confident engagement with recommendations [50]. At the content level, recommendations should be concrete, context-sensitive, explainable, appropriately framed, and sufficiently varied to remain useful over repeated use [46,52]. As contextual constraints may limit the translation from favorable appraisal to enactment, systems should also support feasible alternatives, graded plans, and adaptive revisions when recommendations do not fit users’ time, resources, health status, family responsibilities, or cultural expectations [26,43].

Additionally, the framework can be used as a planning tool for future studies of acceptability and adoption in three ways. First, it can guide pathway testing by helping researchers specify whether they are examining system-to-content pathways, content-to-appraisal pathways, or transitions from appraisal to intention and enactment [46,51]. Second, it can guide mechanism and moderator selection by identifying candidate mechanisms such as perceived fit, actionability, trust calibration, perceived value, and autonomy support, as well as moderators such as input capability, disclosure willingness, AI or technology literacy, verification orientation, and contextual constraints [50,52]. Third, it can guide outcome selection by aligning measures with the perception-intention-enactment pathway, so that studies distinguish users’ appraisal of AI-generated recommendation content, intention to follow it, and actual uptake or enactment. This distinction is important because recommendation-level acceptability and adoption are not equivalent to intervention effectiveness, but they are proximal conditions through which AI-generated lifestyle recommendations can plausibly contribute to downstream behavior change [22,26]. This use of the framework can help future studies move from broad claims about AI acceptance and adoption toward more precise explanations of why users accept or adopt AI-generated lifestyle recommendations.

#### Research Gaps and Future Directions

Several gaps remain across the evidence base. First, outcomes were measured unevenly along the perception-intention-enactment pathway. Proximal perceptions and intentions were common, whereas behavioral enactment was rarely assessed, limiting inference about whether favorable perceptions translate into concrete recommendation adoption [22,26]. Although limited evidence suggests that intention may predict short-term enactment and self-efficacy offers a plausible lens for intention-enactment translation [26], complete pathways linking system- and content-level features to enactment via intermediate psychological mechanisms are rarely specified and empirically tested [40,50]. Second, explicit theoretical grounding was uncommon and was more often used to structure interpretation, content coding, or design derivation than to specify and test causal models [18,27,46,50]. Third, many studies did not report empirically justified sample size decisions or statistical power, limiting confidence in effect estimates and cumulative comparison across studies [26,45]. Finally, recruitment often favored motivated and digitally literate users, which may overestimate acceptability and constrain generalizability [19,26,44].

Future research should strengthen both behavioral outcome measurement and theory development for the acceptability and adoption of AI-generated lifestyle recommendations. Studies should more consistently assess adoption-related outcomes, including intention to follow, in-study uptake, and short-term enactment, ideally combining self-report with objective or trace-based measures, such as accelerometry, logged dietary behaviors, or system-recorded execution markers, to mitigate recall and social desirability biases [59]. In parallel, the field would benefit from theory-driven, testable models integrating behavioral science [60] (eg, COM-B, self-efficacy, persuasion, and behavior change frameworks) with HCI or computing approaches [57], and from designs powered to evaluate hypothesized mechanisms. Broader recruitment and attention to differential capability and context will be important for generalizability [61].

### Limitations of This Review

This review has several limitations. First, variability in study designs, systems, and outcome measures limited direct comparability across studies and precluded quantitative synthesis. Second, inclusions were restricted to English-language records and a defined set of databases, so relevant studies, particularly those in gray literature or evaluations reported outside indexed venues, may have been missed. Third, the narrow eligibility criteria improved interpretability but may limit generalizability to hybrid systems that combine AI-generated recommendations with human review, rule-based safeguards, or predefined expert or guideline content. Fourth, because we restricted inclusion to user-facing systems with real user use, early-stage design-only work was excluded. While this improves ecological relevance, it may underrepresent emerging concepts and novel interaction designs. Together, these scope decisions, alongside the emerging nature of AI-generated lifestyle recommendation research, likely reduced the number of eligible studies and limited the breadth of systems represented. Finally, consistent with the purpose of a scoping review, we mapped and integrated concepts and evidence rather than estimating pooled effects or testing causal pathways. Therefore, the proposed framework should be considered preliminary and evidence-informed and should be refined through future empirical testing.

### Conclusions

This review extends prior AI and digital health reviews by shifting attention from whether AI systems can generate lifestyle advice to whether users accept and adopt the specific recommendations they receive. The synthesis showed that this process is shaped by four linked questions: whether AI systems can generate and deliver recommendations that reflect users’ goals and constraints; whether the resulting content is relevant, actionable, credible, and appropriately framed; whether users can appraise, verify, and engage with the recommendation process; and whether real-world contexts allow recommendations to be taken up or enacted. The integrative framework developed in this review provides a structure for studying these processes across the perception-intention-enactment pathway. It can support future research by clarifying which pathways, mechanisms, moderators, and outcomes should be tested, and can support system design by identifying where recommendation processes may fail. Future work should use more diverse samples, stronger theory-driven designs, and behavioral or trace-based measures to determine when, for whom, and under what conditions AI-generated lifestyle recommendations can become acceptable and adoptable in everyday life. By integrating implementation outcomes, behavioral theory, and human-AI interaction perspectives, this review provides a foundation for moving AI-generated lifestyle recommendations from technically plausible outputs toward user-centered, context-sensitive, and behaviorally actionable support.

## Supplementary material

10.2196/93573 Multimedia Appendix 1 Search strategies, full-text exclusions, and system-characteristic coding.

10.2196/93573 Multimedia Appendix 2 Item-level Mixed Methods Appraisal Tool.

10.2196/93573 Checklist 1 PRISMA-ScR Checklist.

10.2196/93573 Checklist 2 PRISMA-S Search Reporting Details.

## Abbreviations:

AI: artificial intelligence
BCT: behavior change technique
COM-B: Capability, Opportunity, Motivation–Behavior model
HCI: human-computer interaction
JBI: Joanna Briggs Institute
LLM: large language model
MeSH: Medical Subject Headings
MMAT: Mixed Methods Appraisal Tool
NCD: noncommunicable disease
OSF: Open Science Framework
PA: physical activity
PCC: Population, Concept, and Context
PRISMA: Preferred Reporting Items for Systematic Reviews and Meta-Analyses
PRISMA-S: Preferred Reporting Items for Systematic Reviews and Meta-Analyses literature search extension
PRISMA-ScR: Preferred Reporting Items for Systematic Reviews and Meta-Analyses extension for Scoping Reviews
