# Supplementary material for: User Acceptability and Adoption of AI-Generated Lifestyle Intervention Recommendations: Scoping Review and Theoretical Integration
Source: J Med Internet Res. 2026 Jul 14;28:e93573. doi: 10.2196/93573 (PMC13367943; doi:10.2196/93573)
Supplement: Multimedia Appendix 1 [file jmir-v28-e93573-s001.docx]

**Multimedia Appendix**

**Table of Contents**

Full Search Strategies2

Full-Text Exclusions with Reasons26

Detailed system characteristics and feature coding underlying Figure 2.55

**Full Search Strategies**

**1. Ovid MEDLINE(R)**

Database: Ovid MEDLINE(R) ALL

Coverage: 1946 to May 04, 2026

Platform: Ovid

Results: n = 1,007

| # | Search (Ovid syntax) | Results |
| --- | --- | --- |
| 1 | exp Artificial Intelligence/ | 280466 |
| 2 | exp Machine Learning/ | 121643 |
| 3 | exp Natural Language Processing/ | 9275 |
| 4 | ("language model*" or "language models").ti,ab. | 9016 |
| 5 | ("generative ai" or "gen ai" or "generative artificial intelligence").ti,ab. | 2058 |
| 6 | ("large language model*" or LLM* or "foundation model*" or "transformer model*").ti,ab. | 9012 |
| 7 | (GPT or GPT-2 or GPT-3 or GPT-4 or ChatGPT).ti,ab. | 11423 |
| 8 | ("text generat*" or "content generat*" or "natural language generat*").ti,ab. | 545 |
| 9 | (chatbot* or "conversational agent*" or "virtual agent*" or "virtual assistant*" or "digital coach*" or "virtual coach*" or "AI coach*" or "health coach*" or "personalized recommendation*" or "personalised recommendation*" or recommender system*).ti,ab. | 5217 |
| 10 | 1 or 2 or 3 or 4 or 5 or 6 or 7 or 8 or 9 | 293005 |
| 11 | exp Life Style/ | 123562 |
| 12 | exp Health Behavior/ | 392456 |
| 13 | (lifestyle intervention* or lifestyle medicine or "lifestyle psychiatry").ti,ab. | 11053 |
| 14 | ((multi-behavio?r* adj3 intervention*) or multiple lifestyle behavio?r*).ti,ab. | 102 |
| 15 | exp Weight Loss/ | 55836 |
| 16 | exp Weight Reduction Programs/ | 3677 |
| 17 | exp Diet Therapy/ | 68485 |
| 18 | (weight management or weight loss intervention* or diet* or nutrition* or healthy diet*).ti,ab. | 932673 |
| 19 | exp Exercise/ | 286373 |
| 20 | exp Exercise Therapy/ | 75477 |
| 21 | exp Motor Activity/ | 387109 |
| 22 | exp Sports/ | 241423 |
| 23 | exp Yoga/ | 4627 |
| 24 | (exercis* or physical* activ* or sport* or yoga or sedentary behavio?r* or sitting time).ti,ab. | 545689 |
| 25 | exp Sleep Hygiene/ | 6358 |
| 26 | exp Sleep/ | 112798 |
| 27 | (sleep quality or sleep intervention* or sleep hygiene).ti,ab. | 29611 |
| 28 | stress management.ti,ab. | 6590 |
| 29 | exp Stress, Psychological/ | 174819 |
| 30 | (stress management or mindfulness or relaxation training).ti,ab. | 19979 |
| 31 | exp Smoking Cessation/ | 35252 |
| 32 | (smoking cessation or quit smoking).ti,ab. | 31681 |
| 33 | alcohol abstinen*.mp. | 1962 |
| 34 | (alcohol reduction or drinking reduction).ti,ab. | 445 |
| 35 | 11 or 12 or 13 or 14 or 15 or 16 or 17 or 18 or 19 or 20 or 21 or 22 or 23 or 24 or 25 or 26 or 27 or 28 or 29 or 30 or 31 or 32 or 33 or 34 | 2376739 |
| 36 | (accept* or acceptance or acceptability).ti,ab. | 519312 |
| 37 | (adopt* or adoption or uptake).ti,ab. | 736821 |
| 38 | (utiliz* or usage or "actual use").ti,ab. | 895830 |
| 39 | ("intention to use" or "intention to adopt" or "willingness to use" or "continuance intention").ti,ab. | 4356 |
| 40 | (adheren* or compli*).ti,ab. | 1627285 |
| 41 | ((follow* adj3 recommendation*) or (follow* adj3 advice)).ti,ab. | 12253 |
| 42 | (engagement or "user engagement" or receptiv*).ti,ab. | 147324 |
| 43 | (attitude* or preference*).ti,ab. | 366097 |
| 44 | (satisfaction or "user satisfaction" or perceived value).ti,ab. | 184031 |
| 45 | (trust or trustworthiness or credibility or reliability).ti,ab. | 274021 |
| 46 | (transparency or explainab* or interpretab*).ti,ab. | 55550 |
| 47 | ("perceived risk" or "perceived benefit" or privacy concern* or safety concern*).ti,ab. | 25781 |
| 48 | (usability or "user experience" or UX or "perceived usefulness" or "perceived ease of use" or feasibility).ti,ab. | 262409 |
| 49 | 36 or 37 or 38 or 39 or 40 or 41 or 42 or 43 or 44 or 45 or 46 or 47 or 48 | 4454492 |
| 50 | 10 and 35 and 49 | 6029 |
| 51 | (recommend* or advice or prescribe* or prescription* or "treatment recommendation*" or "health recommendation*" or "lifestyle recommendation*" or coaching or "health coach*" or "digital coach*" or "virtual coach*").tw. | 1111794 |
| 52 | 50 and 51 | 1229 |
| 53 | (user* or end-user* or "end user*" or patient* or client* or consumer* or participant* or caregiver* or carer* or parent* or family or families or "target user*" or "intended user*" or "service user*").ti,ab. | 10062033 |
| 54 | 52 and 53 | 1007 |

**2. Embase (Ovid)**

Database: Embase

Coverage: 1974 to May 01, 2026

Platform: Ovid

Results: n = 3,373

| # | Search (Ovid Embase syntax) | Results |
| --- | --- | --- |
| 1 | exp Artificial Intelligence/ | 202809 |
| 2 | exp Machine Learning/ | 745452 |
| 3 | exp Natural Language Processing/ | 21033 |
| 4 | exp Language Model/ | 24495 |
| 5 | ("generative ai" or "gen ai" or "generative artificial intelligence").ti,ab. | 8047 |
| 6 | ("large language model*" or LLM* or "foundation model*" or "transformer model*").ti,ab. | 21011 |
| 7 | (GPT or GPT-2 or GPT-3 or GPT-4 or ChatGPT).ti,ab. | 21910 |
| 8 | ("text generat*" or "content generat*" or "natural language generat*").ti,ab. | 1015 |
| 9 | (chatbot* or "conversational agent*" or "virtual agent*" or "virtual assistant*" or "digital coach*" or "virtual coach*" or "AI coach*" or "health coach*" or "personalized recommendation*" or "personalised recommendation*" or recommender system*).ti,ab. | 11317 |
| 10 | 1 or 2 or 3 or 4 or 5 or 6 or 7 or 8 or 9 | 870467 |
| 11 | exp Life Style/ | 211887 |
| 12 | exp Health Behavior/ | 579461 |
| 13 | (lifestyle intervention* or lifestyle medicine or "lifestyle psychiatry").ti,ab. | 22195 |
| 14 | ((multi-behavio?r* adj3 intervention*) or multiple lifestyle behavio?r*).ti,ab. | 156 |
| 15 | exp Weight Loss/ | 298006 |
| 16 | exp Weight Reduction Program/ | 4709 |
| 17 | exp Diet Therapy/ | 505562 |
| 18 | (weight management or weight loss intervention* or diet* or nutrition* or healthy diet*).ti,ab. | 1457098 |
| 19 | exp Exercise/ | 568495 |
| 20 | exp Exercise Therapy/ | 131300 |
| 21 | exp Physical Activity/ | 685641 |
| 22 | exp Sports/ | 259972 |
| 23 | exp Yoga/ | 16462 |
| 24 | (exercis* or physical* activ* or sport* or yoga or sedentary behavio?r* or sitting time).ti,ab. | 930760 |
| 25 | exp Sleep Hygiene/ | 5135 |
| 26 | exp Sleep/ | 363783 |
| 27 | (sleep quality or sleep intervention* or sleep hygiene).ti,ab. | 65125 |
| 28 | exp Stress Management/ | 10674 |
| 29 | exp Psychological Stress/ | 269348 |
| 30 | (stress management or mindfulness or relaxation training).ti,ab. | 39370 |
| 31 | exp Smoking Cessation/ | 81833 |
| 32 | (smoking cessation or quit smoking).ti,ab. | 52584 |
| 33 | alcohol abstinen*.mp. | 10336 |
| 34 | (alcohol reduction or drinking reduction).ti,ab. | 831 |
| 35 | 11 or 12 or 13 or 14 or (15 or 16 or 17 or 18) or (19 or 20 or 21 or 22 or 23 or 24) or (25 or 26 or 27) or (28 or 29 or 30) or (31 or 32) or (33 or 34) | 4331601 |
| 36 | (accept* or acceptance or acceptability).ti,ab. | 908164 |
| 37 | (adopt* or adoption or uptake).ti,ab. | 1164664 |
| 38 | (utiliz* or usage or "actual use").ti,ab. | 1632334 |
| 39 | ("intention to use" or "intention to adopt" or "willingness to use" or "continuance intention").ti,ab. | 6630 |
| 40 | (adheren* or compli*).ti,ab. | 3046517 |
| 41 | ((follow* adj3 recommendation*) or (follow* adj3 advice)).ti,ab. | 24668 |
| 42 | (engagement or "user engagement" or receptiv*).ti,ab. | 249510 |
| 43 | (attitude* or preference*).ti,ab. | 571513 |
| 44 | (satisfaction or "user satisfaction" or perceived value).ti,ab. | 339420 |
| 45 | (trust or trustworthiness or credibility or reliability).ti,ab. | 462661 |
| 46 | (transparency or explainab* or interpretab*).ti,ab. | 101272 |
| 47 | ("perceived risk" or "perceived benefit" or privacy concern* or safety concern*).ti,ab. | 48026 |
| 48 | (usability or "user experience" or UX or "perceived usefulness" or "perceived ease of use" or feasibility).ti,ab. | 518491 |
| 49 | 36 or 37 or 38 or 39 or 40 or 41 or 42 or 43 or 44 or 45 or 46 or 47 or 48 | 7745737 |
| 50 | 10 and 35 and 49 | 19505 |
| 51 | (recommend* or advice or prescribe* or prescription* or "treatment recommendation*" or "health recommendation*" or "lifestyle recommendation*" or coaching or "health coach*" or "digital coach*" or "virtual coach*").tw. | 2082917 |
| 52 | 50 and 51 | 4010 |
| 53 | (user* or end-user* or "end user*" or patient* or client* or consumer* or participant* or caregiver* or carer* or parent* or family or families or "target user*" or "intended user*" or "service user*").ti,ab. | 17521164 |
| 54 | 52 and 53 | 3373 |

**3. APA PsycInfo (ProQuest)**

Database: APA PsycInfo®

Coverage: 1806 to May 05, 2026

Platform: ProQuest

Results: n = 274

| **Set** | **Search** | **Results** |
| --- | --- | --- |
| S1 | DE.EXACT.EXPLODE("Artificial Intelligence") | 89180 |
| S2 | DE.EXACT.EXPLODE("Machine Learning") | 38598 |
| S3 | DE.EXACT.EXPLODE("Natural Language Processing") | 1848 |
| S4 | TI,AB("language model*" OR "language models") | 2257 |
| S5 | TI,AB("generative ai" OR "gen ai" OR "generative artificial intelligence") | 1325 |
| S6 | TI,AB("large language model*" OR LLM* OR "foundation model*" OR "transformer model*") | 1589 |
| S7 | TI,AB(GPT OR GPT-2 OR GPT-3 OR GPT-4 OR ChatGPT) | 1625 |
| S8 | TI,AB("text generat*" OR "content generat*" OR "natural language generat*") | 463 |
| S9 | TI,AB(chatbot* OR "conversational agent*" OR "virtual agent*" OR "virtual assistant*" OR "digital coach*" OR "virtual coach*" OR "AI coach*" OR "health coach*" OR "personalized recommendation*" OR "personalised recommendation*" OR recommender system*) | 3932 |
| S10 | S1 OR S2 OR S3 OR S4 OR S5 OR S6 OR S7 OR S8 OR S9 | 91873 |
| S11 | DE.EXACT.EXPLODE("Lifestyle") | 16121 |
| S12 | DE.EXACT.EXPLODE("Health Behavior") | 59029 |
| S13 | TI,AB(lifestyle intervention* OR lifestyle medicine OR "lifestyle psychiatry") | 10839 |
| S14 | TI,AB((multi-behavio?r* NEAR/3 intervention*) OR multiple lifestyle behavio?r*) | 1344 |
| S15 | DE.EXACT.EXPLODE("Weight Loss") | 5381 |
| S16 | DE.EXACT("Weight Reduction") | 138 |
| S17 | TI,AB("diet therapy") | 97 |
| S18 | TI,AB(weight management OR weight loss intervention* OR diet* OR nutrition* OR healthy diet*) | 80202 |
| S19 | DE.EXACT.EXPLODE("Exercise") | 37462 |
| S20 | DE.EXACT.EXPLODE("Exercise Therapy") | 847 |
| S21 | TI,AB("Motor Activity") | 6426 |
| S22 | DE.EXACT.EXPLODE("Sports") | 52007 |
| S23 | DE.EXACT.EXPLODE("Yoga") | 3004 |
| S24 | TI,AB(exercis* OR physical* activ* OR sport* OR yoga OR sedentary behavio?r* OR sitting time) | 194017 |
| S25 | TI,AB("Sleep Hygiene") | 1365 |
| S26 | DE.EXACT.EXPLODE("Sleep") | 54939 |
| S27 | TI,AB(sleep quality OR sleep intervention* OR sleep hygiene) | 28813 |
| S28 | TI,AB(stress management) | 16585 |
| S29 | DE.EXACT.EXPLODE("Psychological Stress") | 10417 |
| S30 | TI,AB(stress management OR mindfulness OR relaxation training) | 42111 |
| S31 | DE.EXACT.EXPLODE("Smoking Cessation") | 16266 |
| S32 | TI,AB(smoking cessation OR quit smoking) | 18065 |
| S33 | NOFT(alcohol abstinen*) | 15326 |
| S34 | TI,AB(alcohol reduction OR drinking reduction) | 9335 |
| S35 | S11 OR S12 OR S13 OR S14 OR S15 OR S16 OR S17 OR S18 OR S19 OR S20 OR S21 OR S22 OR S23 OR S24 OR S25 OR S26 OR S27 OR S28 OR S29 OR S30 OR S31 OR S32 OR S33 OR S34 | 482022 |
| S36 | TI,AB(accept* OR acceptance OR acceptability) | 172971 |
| S37 | TI,AB(adopt* OR adoption OR uptake) | 150732 |
| S38 | TI,AB(utiliz* OR usage OR "actual use") | 231848 |
| S39 | TI,AB("intention to use" OR "intention to adopt" OR "willingness to use" OR "continuance intention") | 4785 |
| S40 | TI,AB(adheren* OR compli*) | 101040 |
| S41 | TI,AB((follow* NEAR/3 recommendation*) OR (follow* NEAR/3 advice)) | 3663 |
| S42 | TI,AB(engagement OR "user engagement" OR receptiv*) | 137865 |
| S43 | TI,AB(attitude* OR preference*) | 351716 |
| S44 | TI,AB(satisfaction OR "user satisfaction" OR perceived value) | 163246 |
| S45 | TI,AB(trust OR trustworthiness OR credibility OR reliability) | 174545 |
| S46 | TI,AB(transparency OR explainab* OR interpretab*) | 16639 |
| S47 | TI,AB("perceived risk" OR "perceived benefit" OR privacy concern* OR safety concern*) | 22037 |
| S48 | TI,AB(usability OR "user experience" OR UX OR "perceived usefulness" OR "perceived ease of use" OR feasibility) | 50167 |
| S49 | S36 OR S37 OR S38 OR S39 OR S40 OR S41 OR S42 OR S43 OR S44 OR S45 OR S46 OR S47 OR S48 | 1282055 |
| S50 | S10 AND S35 AND S49 | 1625 |
| S51 | NOFT(recommend* OR advice OR prescribe* OR prescription* OR "treatment recommendation*" OR "health recommendation*" OR "lifestyle recommendation*" OR coaching OR "health coach*" OR "digital coach*" OR "virtual coach*") | 222117 |
| S52 | S50 AND S51 | 332 |
| S53 | TI,AB(user* OR end-user* OR "end user*" OR patient* OR client* OR consumer* OR participant* OR caregiver* OR carer* OR parent* OR family OR families OR "target user*" OR "intended user*" OR "service user*") | 2152418 |
| S54 | S52 AND S53 | 274 |

**4. Web of Science Core Collection**

Database: Web of Science Core Collection

Coverage: 1980 to May 05, 2026

Platform: Web of Science

Editions searched: Science Citation Index Expanded (SCI-EXPANDED) and Social Sciences Citation Index (SSCI)

Results: n = 1,815

Query:

(
 TS=(
 "artificial intelligence"
 OR "machine learning"
 OR "natural language processing"
 OR

"language model*"
 OR "generative ai" OR "gen ai" OR "generative artificial intelligence"
 OR "large

language model*" OR LLM* OR "foundation model*" OR "transformer model*"
 OR GPT OR "GPT-2" OR "GPT-3" OR

"GPT-4" OR ChatGPT
 OR "text generat*" OR "content generat*" OR "natural language generat*"
 OR

chatbot* OR "conversational agent*" OR "virtual agent*" OR "virtual assistant*"
 OR "digital coach*" OR

"virtual coach*" OR "AI coach*" OR "health coach*"
 OR "personalized recommendation*" OR "personalised

recommendation*"
 OR "recommender system*"
 )
)
AND
(
 TS=(
 lifestyle OR "life style*" OR "lifestyle

intervention*" OR "lifestyle medicine" OR "lifestyle psychiatry"
 OR (multi-behavio?r* NEAR/3

intervention*) OR "multiple lifestyle behavio?r*"
 OR "weight loss" OR "weight reduction program*" OR

"weight management"
 OR "weight loss intervention*" OR diet* OR nutrition* OR "healthy diet*"
 OR

exercis* OR "physical* activ*" OR sport* OR yoga
 OR "sedentary behavio?r*" OR "sitting time"
 OR sleep

OR "sleep quality" OR "sleep intervention*" OR "sleep hygiene"
 OR "stress management" OR mindfulness OR

"relaxation training"
 OR "psychological stress"
 OR "smoking cessation" OR "quit smoking"
 OR

"alcohol abstinen*" OR "alcohol reduction" OR "drinking reduction"
 )
)
AND
(
 TS=(
 accept* OR

acceptance OR acceptability
 OR adopt* OR adoption OR uptake
 OR utiliz* OR usage OR "actual use"

OR "intention to use" OR "intention to adopt" OR "willingness to use" OR "continuance intention"
 OR

adheren* OR compli*
 OR (follow* NEAR/3 recommendation*) OR (follow* NEAR/3 advice)
 OR engagement OR

"user engagement" OR receptiv*
 OR attitude* OR preference*
 OR satisfaction OR "user satisfaction" OR

"perceived value"
 OR trust OR trustworthiness OR credibility OR reliability
 OR transparency OR

explainab* OR interpretab*
 OR "perceived risk" OR "perceived benefit" OR "privacy concern*" OR "safety

concern*"
 OR usability OR "user experience" OR UX OR "perceived usefulness" OR "perceived ease of use" OR

feasibility
 )
)
AND
(
 TS=(
 recommend* OR advice OR prescribe* OR prescription*
 OR "treatment

recommendation*"
 OR "health recommendation*"
 OR "lifestyle recommendation*"
 OR coaching OR

"health coach*" OR "digital coach*" OR "virtual coach*"
 )
)
AND
(
 TS=(
 user* OR end-user* OR "end

user*" OR patient* OR client* OR consumer* OR participant*
 OR caregiver* OR carer* OR parent* OR family

OR families
 OR "target user*" OR "intended user*" OR "service user*"
 )
)

**5. Scopus**

Database: Scopus

Coverage: 1788 to May 05, 2026

Platform: Scopus

Results: n = 3,769

Query:

TITLE-ABS-KEY(
 "artificial intelligence" OR "machine learning" OR "natural language processing"
 OR

"language model*"
 OR "generative ai" OR "gen ai" OR "generative artificial intelligence"
 OR "large

language model*" OR LLM* OR "foundation model*" OR "transformer model*"
 OR GPT OR "GPT-2" OR "GPT-3" OR

"GPT-4" OR ChatGPT
 OR "text generat*" OR "content generat*" OR "natural language generat*"
 OR chatbot* OR

"conversational agent*" OR "virtual agent*" OR "virtual assistant*"
 OR "digital coach*" OR "virtual coach*"

OR "AI coach*" OR "health coach*"
 OR "personalized recommendation*" OR "personalised recommendation*"
 OR

"recommender system*"
)
AND
TITLE-ABS-KEY(
 lifestyle OR "life style*" OR "lifestyle intervention*" OR

"lifestyle medicine" OR "lifestyle psychiatry"
 OR (("multi behavior*" OR "multi behaviour*") W/3

intervention*)
 OR ("multiple lifestyle behavior*" OR "multiple lifestyle behaviour*")
 OR "weight loss" OR

"weight reduction program*" OR "weight management" OR "weight loss intervention*"
 OR diet* OR nutrition* OR

"healthy diet*"
 OR exercis* OR "physical activ*" OR sport* OR yoga
 OR ("sedentary behavior*" OR

"sedentary behaviour*") OR "sitting time"
 OR sleep OR "sleep quality" OR "sleep intervention*" OR "sleep

hygiene"
 OR "stress management" OR mindfulness OR "relaxation training"
 OR "psychological stress"
 OR

"smoking cessation" OR "quit smoking"
 OR "alcohol abstinen*" OR "alcohol reduction" OR "drinking

reduction"
)
AND
TITLE-ABS-KEY(
 accept* OR acceptance OR acceptability
 OR adopt* OR adoption OR uptake

OR utiliz* OR usage OR "actual use"
 OR "intention to use" OR "intention to adopt" OR "willingness to use"

OR "continuance intention"
 OR adheren* OR compli*
 OR (follow* W/3 recommendation*) OR (follow* W/3

advice)
 OR engagement OR "user engagement" OR receptiv*
 OR attitude* OR preference*
 OR satisfaction OR

"user satisfaction" OR "perceived value"
 OR trust OR trustworthiness OR credibility OR reliability
 OR

transparency OR explainab* OR interpretab*
 OR "perceived risk" OR "perceived benefit" OR "privacy concern*"

OR "safety concern*"
 OR usability OR "user experience" OR UX OR "perceived usefulness" OR "perceived ease

of use" OR feasibility
)
AND
TITLE-ABS-KEY(
 recommend* OR advice OR prescribe* OR prescription*
 OR

"treatment recommendation*" OR "health recommendation*" OR "lifestyle recommendation*"
 OR coaching OR

"health coach*" OR "digital coach*" OR "virtual coach*"
)
AND
TITLE-ABS-KEY(
 user* OR end-user* OR "end

user*" OR patient* OR client* OR consumer*
 OR participant* OR caregiver* OR carer* OR parent* OR family OR

families
 OR "target user*" OR "intended user*" OR "service user*"
)

**6. ACM Digital Library**

Platform: ACM Digital Library

Coverage: 1908 to May 05, 2026

Platform: ACM Digital Library

Results: n = 1,857

Query:

(
 "generative ai" OR "gen ai" OR "generative artificial intelligence"
 OR large language model* OR llm OR

"foundation model*" OR "transformer model*"
 OR gpt OR "gpt-2" OR "gpt-3" OR "gpt-4" OR chatgpt
 OR

chatbot* OR "conversational agent*" OR "virtual agent*" OR "virtual assistant*"
 OR digital coach* OR

virtual coach* OR "ai coach*" OR "health coach*"
 OR "personalized recommendation*" OR "personalised

recommendation*" OR recommender system*
)
AND
(
 "lifestyle intervention*" OR "lifestyle medicine" OR

"lifestyle psychiatry"
 OR "weight loss" OR "weight reduction program*" OR "weight management" OR "weight

loss intervention*"
 OR diet* OR nutrition* OR "healthy diet*"
 OR exercis* OR "physical activ*" OR sport*

OR yoga
 OR "sedentary behavior*" OR "sedentary behaviour*" OR "sitting time"
 OR sleep OR "sleep quality"

OR "sleep intervention*" OR "sleep hygiene"
 OR "stress management" OR mindfulness OR "relaxation training"

OR "psychological stress"
 OR "smoking cessation" OR "quit smoking"
 OR "alcohol abstinen*" OR "alcohol

reduction" OR "drinking reduction"
)
AND
(
 "treatment recommendation*" OR "health recommendation*" OR

"lifestyle recommendation*"
 OR coaching OR "health coach*" OR "digital coach*" OR "virtual coach*"
)
AND
(

"user acceptance"
 OR "technology acceptance"
 OR "user adoption"
 OR "technology adoption"
 OR

"intention to use"
 OR "perceived usefulness"
 OR "perceived ease of use"
 OR "willingness to use"
 OR

"continuance intention"
 OR "user satisfaction"
 OR "user trust"
)

**7. IEEE Xplore**

Database: IEEE Xplore

Coverage: 1884 to May 05, 2026

Platform: IEEE Xplore

Records retrieved: n = 902

Search string:

(("Artificial Intelligence" OR "Machine Learning" OR "Natural Language Processing" OR "language model" OR "language models" OR "generative ai" OR "generative artificial intelligence" OR "large language model" OR "large language models" OR LLM OR LLMs OR "foundation model" OR "foundation models" OR "transformer model" OR "transformer models" OR GPT OR GPT-2 OR GPT-3 OR GPT-4 OR ChatGPT OR "text generation" OR "content generation" OR "natural language generation" OR chatbot OR "conversational agent" OR "virtual assistant" OR "digital coach" OR "virtual coach" OR "AI coach" OR "health coach" OR "personalized recommendation" OR "recommender system") AND ("Life Style" OR "Health Behavior" OR "lifestyle intervention" OR "lifestyle interventions" OR "lifestyle medicine" OR "lifestyle psychiatry" OR "multi-behaviour intervention" OR "multi-behavior intervention" OR "multiple lifestyle behaviour" OR "Weight Loss" OR "Weight Reduction Programs" OR "Diet Therapy" OR "weight management" OR "weight loss intervention" OR "weight loss interventions" OR diet OR nutrition OR "healthy diet" OR Exercise OR "Exercise Therapy" OR "Motor Activity" OR Sports OR Yoga OR exercise OR "physical activity" OR sport OR yoga OR "sedentary behaviour" OR "sedentary behavior" OR "sitting time" OR "Sleep Hygiene" OR Sleep OR "sleep quality" OR "sleep intervention" OR "sleep interventions" OR "sleep hygiene" OR "stress management" OR "Stress, Psychological" OR mindfulness OR "relaxation training" OR "Smoking Cessation" OR "smoking cessation" OR "quit smoking" OR "alcohol abstinence" OR "alcohol reduction" OR "drinking reduction") AND (accept* OR adoption OR uptake OR utilize OR usage OR "actual use" OR "intention to use" OR "intention to adopt" OR "willingness to use" OR "continuance intention" OR adheren* OR compliance OR (follow NEAR/3 recommendation) OR (follow NEAR/3 advice) OR engagement OR "user engagement" OR receptive OR attitude OR preference OR satisfaction OR "user satisfaction" OR "perceived value" OR trust OR trustworthiness OR credibility OR reliability OR transparency OR explainable OR interpretable OR "perceived risk" OR "perceived benefit" OR "privacy concern" OR "safety concern" OR usability OR "user experience" OR UX OR "perceived usefulness" OR "perceived ease of use" OR feasibility)) AND (recommend* OR advice OR prescribe OR prescription OR "treatment recommendation" OR "health recommendation" OR "lifestyle recommendation" OR coaching OR "health coach" OR "digital coach" OR "virtual coach") AND (user* OR "end user" OR patient OR client OR consumer OR participant OR caregiver OR carer OR parent OR family OR families OR "target user" OR "intended user" OR "service user")

**Full-Text Exclusions with Reasons**

| **No.** | **Excluded studies** | **Primary reason for exclusion** | **No.** | **Excluded studies** | **Primary reason for exclusion** |
| --- | --- | --- | --- | --- | --- |
| 1 | Abdullah, Gaehde [1] | No eligible AI-generated recommendation | 99 | Wang, Song [2] | No eligible AI-generated recommendation |
| 2 | Abdulrahman, Richards [3] | No eligible AI-generated recommendation | 100 | Wang, Hsu [4] | No eligible AI-generated recommendation |
| 3 | Agans, Ma [5] | No eligible AI-generated recommendation | 101 | Weimann, Schlieter [6] | No eligible AI-generated recommendation |
| 4 | Ajovalasit, Attori [7] | No eligible AI-generated recommendation | 102 | van Wissen, Vinkers [8] | No eligible AI-generated recommendation |
| 5 | Albers, Neerincx [9] | No eligible AI-generated recommendation | 103 | Woźniak, Kucharski [10] | No eligible AI-generated recommendation |
| 6 | Albers, Hizli [11] | No eligible AI-generated recommendation | 104 | Yang, Hsieh [12] | No eligible AI-generated recommendation |
| 7 | Albers, Neerincx [13] | No eligible AI-generated recommendation | 105 | Zhang, Kovacs [14] | No eligible AI-generated recommendation |
| 8 | Ali, Rahman [15] | No eligible AI-generated recommendation | 106 | Zhao, Arya [16] | No eligible AI-generated recommendation |
| 9 | Honka, Nieminen [17] | No eligible AI-generated recommendation | 107 | Harikrishnan, Bharathi [18] | No eligible AI-generated recommendation |
| 10 | Aymerich-Franch and Ferrer [19] | No eligible AI-generated recommendation | 108 | Duan [20] | No eligible AI-generated recommendation |
| 11 | Beinema, op den Akker [21] | No eligible AI-generated recommendation | 109 | H, S [22] | No eligible AI-generated recommendation |
| 12 | Beun, Fitrianie [23] | No eligible AI-generated recommendation | 110 | Hotta, Kytö [24] | No eligible AI-generated recommendation |
| 13 | Beun, Both [25] | No eligible AI-generated recommendation | 111 | Sivakumar and Madhumita [26] | No eligible AI-generated recommendation |
| 14 | Bickmore and Picard [27] | No eligible AI-generated recommendation | 112 | Kundu, Ahmed [28] | No eligible AI-generated recommendation |
| 15 | Bohn, Ferrini [29] | No eligible AI-generated recommendation | 113 | M. L, N [30] | No eligible AI-generated recommendation |
| 16 | Buzcu, Pannatier [31] | No eligible AI-generated recommendation | 114 | M. V. K, M [32] | No eligible AI-generated recommendation |
| 17 | Buzcu, Varadhajaran [33] | No eligible AI-generated recommendation | 115 | Ramalingam, Sharma [34] | No eligible AI-generated recommendation |
| 18 | Calvaresi, Calbimonte [35] | No eligible AI-generated recommendation | 116 | Sawad, Althubyani [36] | No eligible AI-generated recommendation |
| 19 | Calvaresi, Eggenschwiler [37] | No eligible AI-generated recommendation | 117 | S, S [38] | No eligible AI-generated recommendation |
| 20 | Cameron, Cameron [39] | No eligible AI-generated recommendation | 118 | Gunawardena, Jayamali [40] | No eligible AI-generated recommendation |
| 21 | Caragiuli, Brunzini [41] | No eligible AI-generated recommendation | 119 | Srivastava, Verma [42] | No eligible AI-generated recommendation |
| 22 | Chauvin, Clavel [43] | No eligible AI-generated recommendation | 120 | Tripathi, Gupta [44] | No eligible AI-generated recommendation |
| 23 | Cheng, Lee [45] | No eligible AI-generated recommendation | 121 | Kandasamy, Ankushavali [46] | No eligible AI-generated recommendation |
| 24 | Chen, Houston [47] | No eligible AI-generated recommendation | 122 | Wen and Feng [48] | No eligible AI-generated recommendation |
| 25 | Chin, Quinn [49] | No eligible AI-generated recommendation | 123 | Priya, Vamsi [50] | Wrong/no acceptability-adoption outcome |
| 26 | Chua, Liu [51] | No eligible AI-generated recommendation | 124 | Bul, Holliday [52] | Wrong/no acceptability-adoption outcome |
| 27 | Coppens, De Pessemier [53] | No eligible AI-generated recommendation | 125 | Anjanamma, Sirisha [54] | Wrong/no acceptability-adoption outcome |
| 28 | Carolis, Palestra [55] | No eligible AI-generated recommendation | 126 | Chang, Wei [56] | Wrong/no acceptability-adoption outcome |
| 29 | Dar, Ekart [57] | No eligible AI-generated recommendation | 127 | Corrò and Chittaro [58] | Wrong/no acceptability-adoption outcome |
| 30 | Davis, Murphy [59] | No eligible AI-generated recommendation | 128 | Kamali, Angelini [60] | Wrong/no acceptability-adoption outcome |
| 31 | De Croon, Segovia-Lizano [61] | No eligible AI-generated recommendation | 129 | Fadhil, Wang [62] | Wrong/no acceptability-adoption outcome |
| 32 | de Kervenoael, Schwob [63] | No eligible AI-generated recommendation | 130 | Gao, Zhang [64] | Wrong/no acceptability-adoption outcome |
| 33 | del Rio, Jimenez [65] | No eligible AI-generated recommendation | 131 | Ghulam, Keegan [66] | Wrong/no acceptability-adoption outcome |
| 34 | Akker, Klaassen [67] | No eligible AI-generated recommendation | 132 | Gupta, Gurbuxani [68] | Wrong/no acceptability-adoption outcome |
| 35 | Nieva, Joaquin [69] | No eligible AI-generated recommendation | 133 | H, K [70] | Wrong/no acceptability-adoption outcome |
| 36 | Dhinagaran and Car [71] | No eligible AI-generated recommendation | 134 | Lithoxoidou, Mastoras [72] | Wrong/no acceptability-adoption outcome |
| 37 | Dino, Dion [73] | No eligible AI-generated recommendation | 135 | Lockwood, Kulkarni [74] | Wrong/no acceptability-adoption outcome |
| 38 | Dubiel, Leiva [75] | No eligible AI-generated recommendation | 136 | Maher, Davis [76] | Wrong/no acceptability-adoption outcome |
| 39 | Dupuy, Sevin [77] | No eligible AI-generated recommendation | 137 | Jhang, Hung [78] | Wrong/no acceptability-adoption outcome |
| 40 | El Majjodi, Starke [79] | No eligible AI-generated recommendation | 138 | Chailaemlak, Trakarnkittikul [80] | Wrong/no acceptability-adoption outcome |
| 41 | El Majjodi, Starke [81] | No eligible AI-generated recommendation | 139 | Mendes Samagaio, Lopes Cardoso [82] | Wrong/no acceptability-adoption outcome |
| 42 | Fasola and Matarić [83] | No eligible AI-generated recommendation | 140 | Shen, Sa [84] | Wrong/no acceptability-adoption outcome |
| 43 | Goldstein, Thomas [85] | No eligible AI-generated recommendation | 141 | Safitri, Mantoro [86] | Wrong/no acceptability-adoption outcome |
| 44 | Görer, Salah [87] | No eligible AI-generated recommendation | 142 | Stein and Brooks [88] | Wrong/no acceptability-adoption outcome |
| 45 | Gutiérrez Hernández, Cardoso [89] | No eligible AI-generated recommendation | 143 | Arum, Genç [90] | Wrong/no acceptability-adoption outcome |
| 46 | Hahn, Rathbun [91] | No eligible AI-generated recommendation | 144 | Wu, Yu [92] | Wrong/no acceptability-adoption outcome |
| 47 | Haug, Boumparis [93] | No eligible AI-generated recommendation | 145 | Yang and Zhao [94] | Wrong/no acceptability-adoption outcome |
| 48 | Hauptmann, Leipold [95] | No eligible AI-generated recommendation | 146 | Nazareth, Kesti [96] | Wrong/no acceptability-adoption outcome |
| 49 | Holkkola, Welling [97] | No eligible AI-generated recommendation | 147 | Davolio, Colarusso [98] | Wrong/no acceptability-adoption outcome |
| 50 | Hors-Fraile, Candel [99] | No eligible AI-generated recommendation | 148 | Varghese and A. M [100] | Wrong/no acceptability-adoption outcome |
| 51 | Jeong, Aymerich-Franch [101] | No eligible AI-generated recommendation | 149 | Yau and Shen [102] | Wrong/no acceptability-adoption outcome |
| 52 | Kanaoka and Mutlu [103] | No eligible AI-generated recommendation | 150 | Sawad, Alakhtar [104] | No eligible system/user evaluation |
| 53 | Kang, Hetrick [105] | No eligible AI-generated recommendation | 151 | Bojic, Ong [106] | No eligible system/user evaluation |
| 54 | Kang and Wei [107] | No eligible AI-generated recommendation | 152 | Buchan, Goel [108] | No eligible system/user evaluation |
| 55 | Kjaerulff, Pedersen [109] | No eligible AI-generated recommendation | 153 | Chew, Achananuparp [110] | No eligible system/user evaluation |
| 56 | Künzler, Mishra [111] | No eligible AI-generated recommendation | 154 | Cuciniello, Amorese [112] | No eligible system/user evaluation |
| 57 | Kowatsch, Lohse [113] | No eligible AI-generated recommendation | 155 | Thom, Ortega [114] | No eligible system/user evaluation |
| 58 | Kumar, Yoo [115] | No eligible AI-generated recommendation | 156 | Figueroa, Torkamaan [116] | No eligible system/user evaluation |
| 59 | Raggioli, Ciccarelli [117] | No eligible AI-generated recommendation | 157 | González-Daza and Feijóo-García [118] | No eligible system/user evaluation |
| 60 | Maharjan, Mendu [119] | No eligible AI-generated recommendation | 158 | Hoang, Chen [120] | No eligible system/user evaluation |
| 61 | Mai, Bauer [121] | No eligible AI-generated recommendation | 159 | Li, Maharjan [122] | No eligible system/user evaluation |
| 62 | Martinho, Crista [123] | No eligible AI-generated recommendation | 160 | Lupetti, Hagens [124] | No eligible system/user evaluation |
| 63 | Mauricio, Flores-Cortegana [125] | No eligible AI-generated recommendation | 161 | Meywirth, Janson [126] | No eligible system/user evaluation |
| 64 | Mauriello, Tantivasadakarn [127] | No eligible AI-generated recommendation | 162 | Meywirth, Janson [128] | No eligible system/user evaluation |
| 65 | Garcia, Mangaba [129] | No eligible AI-generated recommendation | 163 | Rasouli, Ghafurian [130] | No eligible system/user evaluation |
| 66 | Mishra, Hong [131] | No eligible AI-generated recommendation | 164 | Rasouli, Ghafurian [132] | No eligible system/user evaluation |
| 67 | Moore, Al-Tamimi [133] | No eligible AI-generated recommendation | 165 | Rutjes, Willemsen [134] | No eligible system/user evaluation |
| 68 | Moreno-Blanco, Solana-Sánchez [135] | No eligible AI-generated recommendation | 166 | Szymanski, Wimer [136] | No eligible system/user evaluation |
| 69 | Murali, Shamekhi [137] | No eligible AI-generated recommendation | 167 | Torkamaan and Ziegler [138] | No eligible system/user evaluation |
| 70 | Nelekar, Abdulrahman [139] | No eligible AI-generated recommendation | 168 | Tseng, Chen [140] | No eligible system/user evaluation |
| 71 | Oc and Plangger [141] | No eligible AI-generated recommendation | 169 | Arun, Chaudhari [142] | No eligible system/user evaluation |
| 72 | Ono, Yotsuya [143] | No eligible AI-generated recommendation | 170 | Yan, Ren [144] | No eligible system/user evaluation |
| 73 | Park, Chung [145] | No eligible AI-generated recommendation | 171 | Diaz, Carrasco [146] | No eligible system/user evaluation |
| 74 | Patra, Kokkinopoulou [147] | No eligible AI-generated recommendation | 172 | Aharony, Krakovski [148] | No lifestyle recommendation |
| 75 | Paul, Bartmann [149] | No eligible AI-generated recommendation | 173 | Dindorf, Dully [150] | No lifestyle recommendation |
| 76 | Piao, Kim [151] | No eligible AI-generated recommendation | 174 | Herter and Horstmann [152] | No lifestyle recommendation |
| 77 | Putta and Sandbulte [153] | No eligible AI-generated recommendation | 175 | Hoffman, Flom [154] | No lifestyle recommendation |
| 78 | Rabbi, Aung [155] | No eligible AI-generated recommendation | 176 | Kannampallil, Ronneberg [156] | No lifestyle recommendation |
| 79 | Rabbi, Aung [157] | No eligible AI-generated recommendation | 177 | Difini, Martins [158] | No lifestyle recommendation |
| 80 | Radha, Willemsen [159] | No eligible AI-generated recommendation | 178 | Ritschel, Seiderer [160] | No lifestyle recommendation |
| 81 | Rice, Klęczek [161] | No eligible AI-generated recommendation | 179 | Spierling Bagsic, Savin [162] | No lifestyle recommendation |
| 82 | Robinson, Connolly [163] | No eligible AI-generated recommendation | 180 | Sun, Liu [164] | No lifestyle recommendation |
| 83 | Suwan, Temdee [165] | No eligible AI-generated recommendation | 181 | Wu, Summers [166] | No lifestyle recommendation |
| 84 | Shamekhi and Bickmore [167] | No eligible AI-generated recommendation | 182 | Yang, Zheng [168] | No lifestyle recommendation |
| 85 | Shen and Wu [169] | No eligible AI-generated recommendation | 183 | Abbas, Wohn [170] | No lifestyle recommendation |
| 86 | Starke, El Majjodi [171] | No eligible AI-generated recommendation | 184 | McCarren, Eriksson [172] | No lifestyle recommendation |
| 87 | Starke, Musto [173] | No eligible AI-generated recommendation | 185 | Wu, Jiang [174] | No lifestyle recommendation |
| 88 | Stephens, Joerin [175] | No eligible AI-generated recommendation | 186 | Zhang, Hu [176] | No lifestyle recommendation |
| 89 | Sze, Waki [177] | No eligible AI-generated recommendation | 187 | Goulart, Lopes Alves [178] | Abstract only/insufficient text |
| 90 | Thongyoo, Anantapanya [179] | No eligible AI-generated recommendation | 188 | Dinç, Argan [180] | Abstract only/insufficient text |
| 91 | Lara, Cruz [181] | No eligible AI-generated recommendation | 189 | Murphy, Davis [182] | Abstract only/insufficient text |
| 92 | To, Green [183] | No eligible AI-generated recommendation | 190 | Mathews and Jeba [184] | Abstract only/insufficient text |
| 93 | Torkamaan and Ziegler [185] | No eligible AI-generated recommendation | 191 | Hart, Wilson-Barnes [186] | Abstract only/insufficient text |
| 94 | Triantafyllidis, Alexiadis [187] | No eligible AI-generated recommendation | 192 | Ng, Tay [188] | Abstract only/insufficient text |
| 95 | Vandeputte, Herold [189] | No eligible AI-generated recommendation | 193 | Trivedi, Shaw [190] | Abstract only/insufficient text |
| 96 | Vardhan, Hegde [191] | No eligible AI-generated recommendation | 194 | Zhao, Qian [192] | Abstract only/insufficient text |
| 97 | Vemuri, Heintzelman [193] | No eligible AI-generated recommendation | 195 | 194] | Abstract only/insufficient text |
| 98 | V, Thiyagu [195] | No eligible AI-generated recommendation |  |  |  |

1. Abdullah AS, Gaehde S, Bickmore T. A Tablet Based Embodied Conversational Agent to Promote Smoking Cessation among Veterans: A Feasibility Study. Journal of Epidemiology and Global Health. 2018 2018/12/01;8(3):225-30. doi: 10.2991/j.jegh.2018.08.104.

2. Wang X, Song Y, Chen W, Du H, Su X, Wang H. Research and Implementation of Personalized Recommendation Algorithm for Senior Diet Exercise Based on Collaborative Filtering. Proceedings of the 2023 4th International Symposium on Artificial Intelligence for Medicine Science; Chengdu, China: Association for Computing Machinery; 2024. p. 796–804.

3. Abdulrahman A, Richards D, Bilgin AA. Changing users’ health behaviour intentions through an embodied conversational agent delivering explanations based on users’ beliefs and goals. Behaviour & Information Technology. 2023 2023/07/04;42(9):1338-56. doi: 10.1080/0144929X.2022.2073269.

4. Wang Y-F, Hsu M-H, Wang MY-F. HealthLINE: A messaging chatbot for supporting chronic disease self-management. DIGITAL HEALTH. 2025 2025/08/01;11:20552076251394891. doi: 10.1177/20552076251394891.

5. Agans JP, Ma F, Schade S, Sciamanna C. Supporting physical activity adoption through recommender system technology: A pilot study. Journal of Health Psychology. 2025 2025/05/01;30(6):1319-33. doi: 10.1177/13591053241242541.

6. Weimann T, Schlieter H, Fischer M, editors. Designing an Avatar-Based Virtual Coach for Obesity Patients. The Next Wave of Sociotechnical Design; 2021 2021//; Cham: Springer International Publishing.

7. Ajovalasit M, Attori I, Caon M, Salice F, Zhou S, Comai S, editors. Caregiver Acceptability of an LLM-Powered Assistant Interface to Improve Sleep Quality of the Elderly. Computer-Human Interaction Research and Applications; 2025 2025//; Cham: Springer Nature Switzerland.

8. van Wissen A, Vinkers C, van Halteren A, editors. Developing a Virtual Coach for Chronic Patients: A User Study on the Impact of Similarity, Familiarity and Realism. Persuasive Technology; 2016 2016//; Cham: Springer International Publishing.

9. Albers N, Neerincx MA, Aretz NL, Ali M, Ekinci A, Brinkman W-P, editors. Attitudes Toward a Virtual Smoking Cessation Coach: Relationship and Willingness to Continue. Persuasive Technology; 2023 2023//; Cham: Springer Nature Switzerland.

10. Woźniak PW, Kucharski PP, Graaf MMAd, Niess J. Exploring Understandable Algorithms to Suggest Fitness Tracker Goals that Foster Commitment. Proceedings of the 11th Nordic Conference on Human-Computer Interaction: Shaping Experiences, Shaping Society; Tallinn, Estonia: Association for Computing Machinery; 2020. p. Article 35.

11. Albers N, Hizli B, Scheltinga BL, Meijer E, Brinkman W-P. Setting Physical Activity Goals with a Virtual Coach: Vicarious Experiences, Personalization and Acceptance. Journal of Medical Systems. 2023 2023/01/30;47(1):15. doi: 10.1007/s10916-022-01899-9.

12. Yang L, Hsieh C-K, Yang H, Pollak JP, Dell N, Belongie S, et al. Yum-Me: A Personalized Nutrient-Based Meal Recommender System. ACM Trans Inf Syst. 2017;36(1):Article 7. doi: 10.1145/3072614.

13. Albers N, Neerincx MA, Penfornis KM, Brinkman W-P. Users’ needs for a digital smoking cessation application and how to address them: a mixed-methods study. PeerJ. 2022;10:e13824. doi: 10.7717/peerj.13824.

14. Zhang AW, Kovacs C, Pablo LD, Zhang J, Bai M, Jeong S, et al., editors. Exploring Robot Personality Traits and Their Influence on User Affect and Experience. 2025 20th ACM/IEEE International Conference on Human-Robot Interaction (HRI); 2025 4-6 March 2025.

15. Ali SH, Rahman F, Kuwar A, Khanna T, Nayak A, Sharma P, et al. Rapid, Tailored Dietary and Health Education Through A Social Media Chatbot Microintervention: Development and Usability Study With Practical Recommendations. JMIR Form Res. 2024 2024/12/9;8:e52032. doi: 10.2196/52032.

16. Zhao Z, Arya A, Orji R, Chan G. Effects of a Personalized Fitness Recommender System Using Gamification and Continuous Player Modeling: System Design and Long-Term Validation Study. JMIR Serious Games. 2020;8(4):e19968. PMID: 33200994. doi: 10.2196/19968.

17. Honka AM, Nieminen H, Similä H, Kaartinen J, Gils MV. A Comprehensive User Modeling Framework and a Recommender System for Personalizing Well-Being Related Behavior Change Interventions: Development and Evaluation. IEEE Access. 2022;10:116766-83. doi: 10.1109/ACCESS.2022.3218776.

18. Harikrishnan U, Bharathi SS, Athisaiyaraj S, Thangaraj P, Arunachalavadivu P, Anto AC, editors. AI-Enhanced Fitness Coaching: Personalized Workout Plans with Integrated IoT Data and Deep Learning. 2025 9th International Conference on Electronics, Communication and Aerospace Technology (ICECA); 2025 5-7 Nov. 2025.

19. Aymerich-Franch L, Ferrer I. Investigating the use of speech-based conversational agents for life coaching. International Journal of Human-Computer Studies. 2022 2022/03/01/;159:102745. doi: 10.1016/j.ijhcs.2021.102745.

20. Duan H, editor. Data Mining-Driven Decision Support System for Sports Training Pattern Recognition and Optimization. 2025 4th International Conference on Artificial Intelligence and Computer Information Technology (AICIT); 2025 19-21 Sept. 2025.

21. Beinema T, op den Akker H, Hurmuz M, Jansen-Kosterink S, Hermens H. Automatic topic selection for long-term interaction with embodied conversational agents in health coaching: A micro-randomized trial. Internet Interventions. 2022 2022/03/01/;27:100502. doi: 10.1016/j.invent.2022.100502.

22. H KG, S N, A. K BH, K R, K DN, P G, editors. An AI-Powered Personalized Fitness Recommendation System Using TF-IDF and Gemini Models. 2025 International Conference on Intelligent Systems and Pioneering Innovations in Robotics and Electric Mobility (INSPIRE); 2025 20-21 Nov. 2025.

23. Beun RJ, Fitrianie S, Griffioen-Both F, Spruit S, Horsch C, Lancee J, et al. Talk and Tools: the best of both worlds in mobile user interfaces for E-coaching. Personal and Ubiquitous Computing. 2017 2017/08/01;21(4):661-74. doi: 10.1007/s00779-017-1021-5.

24. Hotta S, Kytö M, Koivusalo S, Heinonen S, Marttinen P. Personalized Glucose Management With AI: Pilot Study Using a Multiarmed Bandit Approach. JMIR Form Res. 2026 2026/3/19;10:e70826. doi: 10.2196/70826.

25. Beun RJ, Both F, Lancee J, editors. Negotiation in automated e-coaching: an application in mobile insomnia treatment. Proceedings of the 32nd International BCS Human Computer Interaction Conference (HCI 2018); 2018: BCS Learning and Development Ltd.

26. Sivakumar KS, Madhumita G, editors. Design of an AI-Based Decision Support System for Stress Reduction and Workplace Spirituality Enhancement in Educational Institutions. 2025 IEEE 5th International Conference on ICT in Business Industry & Government (ICTBIG); 2025 12-13 Dec. 2025.

27. Bickmore TW, Picard RW. Establishing and maintaining long-term human-computer relationships. ACM Trans Comput-Hum Interact. 2005;12(2):293–327. doi: 10.1145/1067860.1067867.

28. Kundu RK, Ahmed I, Hoque KA, editors. PILAR: Personalizing Augmented Reality Interactions with LLM-based Human-Centric and Trustworthy Explanations for Daily Use Cases. 2025 IEEE International Symposium on Mixed and Augmented Reality Adjunct (ISMAR-Adjunct); 2025 8-12 Oct. 2025.

29. Bohn T, Ferrini K, Stahl C. LIFANA – toward developing a meal recommender system as a dietary support app for the elderly: Evaluation in field trials, pitfalls and lessons learned, and the path forward: Hogrefe AG; 2024. 221–38 p. ISBN: 0300-9831.

30. M. L R, N P, V S, editors. Al-Powered Fitness and Diet Recommendation System with Integrated Conversational Chatbot. 2026 6th International Conference on Expert Clouds and Applications (ICOECA); 2026 9-11 March 2026.

31. Buzcu B, Pannatier Y, Aydoğan R, Ignaz Schumacher M, Calbimonte J-P, Calvaresi D, editors. A Framework for Explainable Multi-purpose Virtual Assistants: A Nutrition-Focused Case Study. Explainable and Transparent AI and Multi-Agent Systems; 2024 2024//; Cham: Springer Nature Switzerland.

32. M. V. K P, M N, V P, V V, V R, editors. Comparative Performance Analysis of Machine Learning Models for Health and Nutrition Prediction. 2026 5th International Conference on Communication, Computing and Electronics Systems (ICCCES); 2026 21-23 Jan. 2026.

33. Buzcu B, Varadhajaran V, Tchappi I, Najjar A, Calvaresi D, Aydoğan R, editors. Explanation-Based Negotiation Protocol for Nutrition Virtual Coaching. PRIMA 2022: Principles and Practice of Multi-Agent Systems; 2023 2023//; Cham: Springer International Publishing.

34. Ramalingam S, Sharma R, Deshpande SV, Kaur M, Ashok T, Khan AA, editors. Explainable AI for Holistic Wellness Planning: Transparent Personalization of Exercise, Diet, and Mental Health Routines. 2025 International Conference on Emerging Trends in Networks and Computer Communications (ETNCC); 2025 5-7 Aug. 2025.

35. Calvaresi D, Calbimonte J-P, Siboni E, Eggenschwiler S, Manzo G, Hilfiker R, et al. EREBOTS: Privacy-Compliant Agent-Based Platform for Multi-Scenario Personalized Health-Assistant Chatbots. Electronics [Internet]. 2021; 10(6):[666 p.].

36. Sawad AB, Althubyani M, Alharbi SA, Alorini A, Jefry W, Alotaibi NN, et al., editors. Advancing Prediabetes Care with Personalised Conversational Agents. 2025 IEEE International Conference on E-Business Engineering (ICEBE); 2025 10-12 Nov. 2025.

37. Calvaresi D, Eggenschwiler S, Calbimonte J-P, Manzo G, Schumacher M. A personalized agent-based chatbot for nutritional coaching. IEEE/WIC/ACM International Conference on Web Intelligence and Intelligent Agent Technology; Melbourne, VIC, Australia: Association for Computing Machinery; 2022. p. 682–7.

38. S S, S P, Priscilla R, Patturose JGB, editors. Smart Diet Planner:An AI-Powered Web Application for Personalized Nutrition Management. 2025 International Conference on Future Technologies (ICFT); 2025 7-8 Nov. 2025.

39. Cameron G, Cameron D, Megaw G, Bond R, Mulvenna M, O’Neill S, et al., editors. Assessing the Usability of a Chatbot for Mental Health Care. Internet Science; 2019 2019//; Cham: Springer International Publishing.

40. Gunawardena SNA, Jayamali PLW, Methsandi KAN, Gamage RPDD, Wijendra D, Krishara J, editors. FITGEN – AI Smart Fitness Companion: An Integrated Multi-Modal Approach to Personalized Health and Fitness Management. 2025 7th International Conference on Advancements in Computing (ICAC); 2025 9-10 Dec. 2025.

41. Caragiuli M, Brunzini A, Massera C, Candelari M, Germani M. Assistive Robots for Older Adults: A Human-Robot Interaction Study. Proceedings of the 18th ACM International Conference on PErvasive Technologies Related to Assistive Environments: Association for Computing Machinery; 2025. p. 46–59.

42. Srivastava A, Verma D, Singh KK, Sharma VS, editors. Real Time Fitness and Diet Tracking System. 2025 IEEE 7th International Conference on Computing, Communication and Automation (ICCCA); 2025 28-30 Nov. 2025.

43. Chauvin R, Clavel C, Sabouret N, Ravenet B. A virtual coach with more or less empathy: impact on older adults' engagement to exercise. Proceedings of the 23rd ACM International Conference on Intelligent Virtual Agents; Würzburg, Germany: Association for Computing Machinery; 2023. p. Article 2.

44. Tripathi P, Gupta N, Kahol K, editors. Reinforcement Learning-Driven Nutrition Coaching. 2025 IEEE International Conference on Collaborative Advances in Software and COmputiNg (CASCON); 2025 10-13 Nov. 2025.

45. Cheng W-H, Lee Y-C, Jamieson J, Wang W-H, Lin W-C. Understanding How Chatbot Phrasing Styles and Care Demonstration Influence Overweight Users' Adherence Intention Towards Chatbots Supporting Weight Management. Proc ACM Hum-Comput Interact. 2025;9(7):Article CSCW336. doi: 10.1145/3757517.

46. Kandasamy U, Ankushavali M, Asmita, Nagar S, Chinnaiyan R, Sharma P, editors. A Comprehensive Study on Machine Learning-Based Optimization and Personalization of Gym Workouts. 2025 IEEE 1st International Conference on Smart Innovations in Systems, Infrastructure, Mechanical, Power, AI and Computing Technologies (SISIMPACT); 2025 28-29 Nov. 2025.

47. Chen J, Houston TK, Faro JM, Nagawa CS, Orvek EA, Blok AC, et al. Evaluating the use of a recommender system for selecting optimal messages for smoking cessation: patterns and effects of user-system engagement. BMC Public Health. 2021 2021/09/26;21(1):1749. doi: 10.1186/s12889-021-11803-8.

48. Wen Y, Feng T. Computer-Based Motion Analysis and Personalized Training Recommendation for Aerobics and Yoga Exercises. Journal of Applied Science and Engineering. 2026;32:1-9. doi: 10.6180/jase.202609_32.009.

49. Chin J, Quinn K, Muramatsu N, Marquez D. A User Study on the Feasibility and Acceptance of Delivering Physical Activity Programs to Older Adults through Conversational Agents. Proceedings of the Human Factors and Ergonomics Society Annual Meeting. 2020 2020/12/01;64(1):33-7. doi: 10.1177/1071181320641010.

50. Priya BH, Vamsi B, Reddy AA, Radhika M, Hariharan S, Kekreja V, editors. Speech enabled personal workout assistant recommendation system. 2024 International Conference on Innovations and Challenges in Emerging Technologies (ICICET); 2024 7-8 June 2024.

51. Chua AJ, Liu S, Zhang H, Fan X, editors. A Persuasive Chatbot in an Aging-in-Place Environment. Intelligent Autonomous Systems 18; 2024 2024//; Cham: Springer Nature Switzerland.

52. Bul K, Holliday N, Bhuiyan MRA, Clark CCT, Allen J, Wark PA. Usability and Preliminary Efficacy of an Artificial Intelligence–Driven Platform Supporting Dietary Management in Diabetes: Mixed Methods Study. JMIR Hum Factors. 2023 2023/8/9;10:e43959. doi: 10.2196/43959.

53. Coppens I, De Pessemier T, Martens L. Investigating different recommender algorithms in the domain of physical activity recommendations: a longitudinal between-subjects user study. User Modeling and User-Adapted Interaction. 2025 2025/02/18;35(1):6. doi: 10.1007/s11257-025-09427-3.

54. Anjanamma C, Sirisha G, Sravani B, Shilpa K, Narayana CVL, Vivekanandhan V, editors. Personalized Food Nutrient Recommendations for Kids using AI and Behavior Analysis. 2024 9th International Conference on Communication and Electronics Systems (ICCES); 2024 16-18 Dec. 2024.

55. Carolis BND, Palestra G, Oranger E. Social Robots vs. Chatbots: Evaluating the Effect as a Persuasive Technology for Children in the Healthy Eating Domain. Proceedings of the 2024 International Conference on Advanced Visual Interfaces; Arenzano, Genoa, Italy: Association for Computing Machinery; 2024. p. Article 74.

56. Chang CC, Wei CH, Hsiao S, Yang CHT, editors. Meal Image Recognition and Healthy Meal Combination Recommendation System Integrated with Generative Artificial Intelligence. 2024 IEEE 6th Eurasia Conference on Biomedical Engineering, Healthcare and Sustainability (ECBIOS); 2024 14-16 June 2024.

57. Dar S, Ekart A, Bernardet U. Usability, acceptance, and the role of realism in virtual humans for breathing exercise training. Scientific Reports. 2025 2025/01/09;15(1):1536. doi: 10.1038/s41598-024-82886-7.

58. Corrò C, Chittaro L, editors. Exploring the Potential and Limitations of Large Language Models to Control the Behavior of Embodied Persuasive Agents. Persuasive Technology; 2025 2025//; Cham: Springer Nature Switzerland.

59. Davis CR, Murphy KJ, Curtis RG, Maher CA. A Process Evaluation Examining the Performance, Adherence, and Acceptability of a Physical Activity and Diet Artificial Intelligence Virtual Health Assistant. International Journal of Environmental Research and Public Health [Internet]. 2020; 17(23):[9137 p.].

60. Kamali ME, Angelini L, Caon M, Andreoni G, Khaled OA, Mugellini E. Towards the NESTORE e-Coach: a Tangible and Embodied Conversational Agent for Older Adults. Proceedings of the 2018 ACM International Joint Conference and 2018 International Symposium on Pervasive and Ubiquitous Computing and Wearable Computers; Singapore, Singapore: Association for Computing Machinery; 2018. p. 1656–63.

61. De Croon R, Segovia-Lizano D, Finglas P, Vanden Abeele V, Verbert K. An Explanation Interface for Healthy Food Recommendations in a Real-Life Workplace Deployment: User-Centered Design Study. JMIR Mhealth Uhealth. 2025 2025/2/11;13:e51271. doi: 10.2196/51271.

62. Fadhil A, Wang Y, Reiterer H. Assistive Conversational Agent for Health Coaching: A Validation Study. Methods Inf Med. 2019 2019/07/05;58(01):009-23. doi: 10.1055/s-0039-1688757.

63. de Kervenoael R, Schwob A, Hasan R, Kemari S. Food choice and the epistemic value of the consumption of recommender systems: the case of Yuka’s perceived value in France. Behaviour & Information Technology. 2024 2024/05/18;43(7):1381-400. doi: 10.1080/0144929X.2023.2212088.

64. Gao Y, Zhang J, He Z, Zhou Z. Feasibility and Usability of an Artificial Intelligence—Powered Gamification Intervention for Enhancing Physical Activity Among College Students: Quasi-Experimental Study. JMIR Serious Games. 2025 2025/3/24;13:e65498. doi: 10.2196/65498.

65. del Rio A, Jimenez J, Medina-García R, Lozano-Hernández C, Alvarez F, Serrano J. Improving Quality of Life in Chronic Patients: A Pilot Study on the Effectiveness of a Health Recommender System and Its Usability. Applied Sciences [Internet]. 2023; 13(10):[5850 p.].

66. Ghulam H, Keegan B, Ross R, editors. Active Listening in Virtual Interactive Coaching: Prompt Strategies and User Assessment. 38th International BCS Human-Computer Interaction Conference; 2025: BCS Learning & Development.

67. Akker Rod, Klaassen R, Bul K, Kato PM, Burg G-Jvd, Bitonto Pd. Let them play: experiences in the wild with a gamification and coaching system for young diabetes patients. Proceedings of the 11th EAI International Conference on Pervasive Computing Technologies for Healthcare; Barcelona, Spain: Association for Computing Machinery; 2017. p. 409–18.

68. Gupta L, Gurbuxani S, Madan K. Virtual Fitness Trainer using Artificial Intelligence. Proceedings of the 2024 Sixteenth International Conference on Contemporary Computing; Noida, India: Association for Computing Machinery; 2024. p. 226–33.

69. Nieva JOD, Joaquin JA, Tan CB, Te RKM, Ong E. Investigating Students’ Use of a Mental Health Chatbot to Alleviate Academic Stress. 6th International ACM In-Cooperation HCI and UX Conference; Jakarta & Bandung, Indonesia: Association for Computing Machinery; 2021. p. 1–10.

70. H N, K S, S. P G, S G, editors. NUTRIC AI: an AI-Assisted Personalized Nutrition Recommendation System. 2025 11th International Conference on Communication and Signal Processing (ICCSP); 2025 5-7 June 2025.

71. Dhinagaran DA, Car LT. Public perceptions of a healthy lifestyle change conversational agent in Singapore: A qualitative study. DIGITAL HEALTH. 2022 2022/01/01;8:20552076221131190. doi: 10.1177/20552076221131190.

72. Lithoxoidou E-E, Mastoras R-E, Papaprodromou A, Georgiadis C, Jimenez PA, Gonzalez S, et al., editors. A Virtual Coach and a Worker Dashboard to Promote Well-Being and Workability: An Acceptance Study. Universal Access in Human-Computer Interaction Novel Design Approaches and Technologies; 2022 2022//; Cham: Springer International Publishing.

73. Dino MJS, Dion KW, Abadir PM, Budhathoki C, Huang C-M, Padula WV, et al. What drives older adults’ acceptance of virtual humans? A conjoint and latent class analysis on virtual exercise coach attributes for a community-based exercise program. Computers in Human Behavior. 2025 2025/03/01/;164:108507. doi: https://doi.org/10.1016/j.chb.2024.108507.

74. Lockwood KG, Kulkarni PR, Paruthi J, Buch LS, Chaffard M, Schitter EC, et al. Evaluating a New Digital App–Based Program for Heart Health: Feasibility and Acceptability Pilot Study. JMIR Form Res. 2024 2024/5/24;8:e50446. doi: 10.2196/50446.

75. Dubiel M, Leiva LA, Bongard-Blanchy K, Sergeeva A. “Hey Genie, You Got Me Thinking about My Menu Choices!” Impact of Proactive Feedback on User Perception and Reflection in Decision-making Tasks. ACM Trans Comput-Hum Interact. 2024;31(5):Article 56. doi: 10.1145/3685274.

76. Maher CA, Davis CR, Curtis RG, Short CE, Murphy KJ. A Physical Activity and Diet Program Delivered by Artificially Intelligent Virtual Health Coach: Proof-of-Concept Study. JMIR Mhealth Uhealth. 2020 2020/7/10;8(7):e17558. doi: 10.2196/17558.

77. Dupuy L, Sevin Ed, Micoulaud-Franchi J-A, Philip P. Factors associated with acceptance of a virtual companion providing screening and advices for sleep problems during COVID-19 crisis. Proceedings of the 21st ACM International Conference on Intelligent Virtual Agents; Virtual Event, Japan: Association for Computing Machinery; 2021. p. 48–51.

78. Jhang NW, Hung YH, Lin YC, Wu YH, editors. Applying Machine Learning to Design and Evaluate White Noise Recommendation System for Insomniacs. 2020 IEEE Eurasia Conference on IOT, Communication and Engineering (ECICE); 2020 23-25 Oct. 2020.

79. El Majjodi A, Starke AD, Elahi M, Trattner C, editors. The Interplay between Food Knowledge, Nudges, and Preference Elicitation Methods Determines the Evaluation of a Recipe Recommender System. IntRS@ RecSys; 2023.

80. Chailaemlak P, Trakarnkittikul P, Wattano S, Tarnpradab S, editors. PlatePal: A Comprehensive Image-Based Food Analysis and Dietary Assistant Mobile Application. 2024 International Conference on Electrical, Communication and Computer Engineering (ICECCE); 2024 30-31 Oct. 2024.

81. El Majjodi A, Starke A, Trattner C, Petruzzelli A, Musto C. Nudging Healthy Choices: Leveraging LLM-Generated Hashtags and Explanations in Personalized Food Recommendations. 2025.

82. Mendes Samagaio Á, Lopes Cardoso H, Ribeiro D, editors. A Chatbot for Recipe Recommendation and Preference Modeling. Progress in Artificial Intelligence; 2021 2021//; Cham: Springer International Publishing.

83. Fasola J, Matarić MJ. A socially assistive robot exercise coach for the elderly. J Hum-Robot Interact. 2013;2(2):3–32. doi: 10.5898/JHRI.2.2.Fasola.

84. Shen Y, Sa HJ, Han J-H. Continued use of artificial intelligence coaching services: Application of the value-based acceptance model. Social Behavior and Personality: an international journal. 2024;52(9):1-15. doi: 10.2224/sbp.13493.

85. Goldstein SP, Thomas JG, Foster GD, Turner-McGrievy G, Butryn ML, Herbert JD, et al. Refining an algorithm-powered just-in-time adaptive weight control intervention: A randomized controlled trial evaluating model performance and behavioral outcomes. Health Informatics Journal. 2020 2020/12/01;26(4):2315-31. doi: 10.1177/1460458220902330.

86. Safitri S, Mantoro T, Bhakti MAC, Wandy W, editors. Cooking and Food Information Chatbot System using GPT-3. 2023 IEEE 9th International Conference on Computing, Engineering and Design (ICCED); 2023 7-8 Nov. 2023.

87. Görer B, Salah AA, Akın HL. An autonomous robotic exercise tutor for elderly people. Autonomous Robots. 2017 2017/03/01;41(3):657-78. doi: 10.1007/s10514-016-9598-5.

88. Stein N, Brooks K. A Fully Automated Conversational Artificial Intelligence for Weight Loss: Longitudinal Observational Study Among Overweight and Obese Adults. JMIR Diabetes. 2017 2017/11/01;2(2):e28. doi: 10.2196/diabetes.8590.

89. Gutiérrez Hernández F, Cardoso B, Verbert K, Elsweiler D, Hors-Fraile S, Ludwig B, et al., editors. Phara: A personal health augmented reality assistant to support decision-making at grocery stores. Proceedings of the International Workshop on Health Recommender Systems co-located with ACM RecSys 2017; 2017: R. Piskac c/o Redaktion Sun SITE Informatik V RWTH Aachen.

90. Arum Sv, Genç HU, Reidsma D, Karahanoğlu A. Selective Trust: Understanding Human-AI Partnerships in Personal Health Decision-Making Process. Proceedings of the 2025 CHI Conference on Human Factors in Computing Systems: Association for Computing Machinery; 2025. p. Article 1026.

91. Hahn L, Rathbun SL, Schmidt MD, Johnsen K, Annesi JJ, Ahn SJ. Using Virtual Agents and Activity Monitors to Autonomously Track and Assess Self-Determined Physical Activity Among Young Children: A 6-Week Feasibility Field Study. Cyberpsychology, Behavior, and Social Networking. 2020 2020/07/01;23(7):471-8. doi: 10.1089/cyber.2019.0491.

92. Wu IY, Yu Y, Cheng S-J, Tu W-J, Sung T-J. Acceptance and sustainability of health promotion solutions for the elderly in Taiwan: evidence from SHI-LIN elderly university in Taipei. Proceedings of the 12th ACM International Conference on PErvasive Technologies Related to Assistive Environments; Rhodes, Greece: Association for Computing Machinery; 2019. p. 21–7.

93. Haug S, Boumparis N, Wenger A, Paz Castro R, Schaub MP. Mobile App-Based Coaching for Alcohol Prevention among Adolescents: Pre–Post Study on the Acceptance and Effectiveness of the Program “MobileCoach Alcohol”. International Journal of Environmental Research and Public Health [Internet]. 2023; 20(4):[3263 p.].

94. Yang Y, Zhao Y. Personalized Sports Health Recommendation System Assisted by Q-Learning Algorithm. International Journal of Human–Computer Interaction. 2025 2025/02/16;41(4):1889-901. doi: 10.1080/10447318.2023.2295693.

95. Hauptmann H, Leipold N, Madenach M, Wintergerst M, Lurz M, Groh G, et al. Effects and challenges of using a nutrition assistance system: results of a long-term mixed-method study. User Modeling and User-Adapted Interaction. 2022 2022/11/01;32(5):923-75. doi: 10.1007/s11257-021-09301-y.

96. Nazareth P, Kesti S, Nayak A, M G, P. K A, editors. SleepSensei-AI: Revolutionizing Sleep Coaching. 2025 International Conference on Intelligent Computing, Information and Control Systems (ICOIICS); 2025 19-21 Nov. 2025.

97. Holkkola M, Welling J, Frank L. How Are Digital Coaches’ Anthropomorphic Features Experienced by Young Men? Adopting a Digital Coach to Increase Exercise and Reduce Sitting. 2025. doi: 10.24251/HICSS.2025.097.

98. Davolio N, Colarusso F, Yuksel S, Casaccia S, editors. Personalized Multi-Agent Recommendation System for Monitoring and Coaching through Wearable and Non-Invasive Sensors. 2025 IEEE International Conference on Metrology for eXtended Reality, Artificial Intelligence and Neural Engineering (MetroXRAINE); 2025 22-24 Oct. 2025.

99. Hors-Fraile S, Candel MJJM, Schneider F, Malwade S, Nunez-Benjumea FJ, Syed-Abdul S, et al. Applying Collective Intelligence in Health Recommender Systems for Smoking Cessation: A Comparison Trial. Electronics [Internet]. 2022; 11(8):[1219 p.].

100. Varghese C, A. M K, editors. SnapCook: AI-Powered Real-Time Ingredient Detection and AR-based Cooking Assistant. 2026 International Conference on Electronics and Renewable Systems (ICEARS); 2026 11-13 Feb. 2026.

101. Jeong S, Aymerich-Franch L, Arias K, Alghowinem S, Lapedriza A, Picard R, et al. Deploying a robotic positive psychology coach to improve college students’ psychological well-being. User Modeling and User-Adapted Interaction. 2023 2023/04/01;33(2):571-615. doi: 10.1007/s11257-022-09337-8.

102. Yau Y, Shen YC. “Though helpful, still hesitant”: a TAM-based qualitative study on older adults’ ambivalent acceptance and model extensions in AI fitness coaches. Frontiers in Psychology. 2025 2025-December-17;Volume 16 - 2025. doi: 10.3389/fpsyg.2025.1666755.

103. Kanaoka T, Mutlu B. Designing a Motivational Agent for Behavior Change in Physical Activity. Proceedings of the 33rd Annual ACM Conference Extended Abstracts on Human Factors in Computing Systems; Seoul, Republic of Korea: Association for Computing Machinery; 2015. p. 1445–50.

104. Sawad AB, Alakhtar R, Alturki B, Narayan B, Lin S, Prasad M, et al. Towards a Design Framework for Conversational Agents for Diabetes Prevention. Proceedings of the 35th Australian Computer-Human Interaction Conference; Wellington, New Zealand: Association for Computing Machinery; 2024. p. 387–98.

105. Kang A, Hetrick S, Cargo T, Hopkins S, Ludin N, Bodmer S, et al. Exploring Young Adults’ Views About Aroha, a Chatbot for Stress Associated With the COVID-19 Pandemic: Interview Study Among Students. JMIR Form Res. 2023 2023/10/12;7:e44556. doi: 10.2196/44556.

106. Bojic I, Ong QC, Ito S, Liu J, Lawate A, Palaiyan M, et al. AI-empowered health coaching for university students: A mixed-method process evaluation. Computers in Biology and Medicine. 2025 2025/08/01/;194:110271. doi: 10.1016/j.compbiomed.2025.110271.

107. Kang J, Wei L. "Give Me the Support I Want!": The Effect of Matching an Embodied Conversational Agent's Social Support to Users' Social Support Needs in Fostering Positive User-Agent Interaction. Proceedings of the 6th International Conference on Human-Agent Interaction; Southampton, United Kingdom: Association for Computing Machinery; 2018. p. 106–13.

108. Buchan ML, Goel K, Schneider CK, Steullet V, Bratton S, Basch E. National implementation of an artificial intelligence–based virtual dietitian for patients with cancer. JCO clinical cancer informatics. 2024;8:e2400085. doi: 10.1200/CCI.24.00085.

109. Kjaerulff SB, Pedersen SB, Sigvardsen TJ, Berkel Nv, Papachristos E. Exploring VUI-Supported Mindfulness Techniques for Smoking Cessation. Proceedings of the 6th ACM Conference on Conversational User Interfaces; Luxembourg, Luxembourg: Association for Computing Machinery; 2024. p. Article 1.

110. Chew HSJ, Achananuparp P, Dalakoti M, Chew NWS, Chin YH, Gao Y, et al. Public acceptance of using artificial intelligence-assisted weight management apps in high-income southeast Asian adults with overweight and obesity: a cross-sectional study. Frontiers in Nutrition. 2024 2024-February-07;Volume 11 - 2024. doi: 10.3389/fnut.2024.1287156.

111. Künzler F, Mishra V, Kramer J-N, Kotz D, Fleisch E, Kowatsch T. Exploring the State-of-Receptivity for mHealth Interventions. Proc ACM Interact Mob Wearable Ubiquitous Technol. 2020;3(4):Article 140. doi: 10.1145/3369805.

112. Cuciniello M, Amorese T, Greco C, Raimo G, Cordasco G, Kornes MS, et al., editors. A cross-cultural survey to identify Seniors’ preferences towards the Empathic Virtual Coach. 2022 IEEE Intl Conf on Dependable, Autonomic and Secure Computing, Intl Conf on Pervasive Intelligence and Computing, Intl Conf on Cloud and Big Data Computing, Intl Conf on Cyber Science and Technology Congress (DASC/PiCom/CBDCom/CyberSciTech); 2022 12-15 Sept. 2022.

113. Kowatsch T, Lohse K-M, Erb V, Schittenhelm L, Galliker H, Lehner R, et al. Hybrid Ubiquitous Coaching With a Novel Combination of Mobile and Holographic Conversational Agents Targeting Adherence to Home Exercises: Four Design and Evaluation Studies. J Med Internet Res. 2021 2021/2/22;23(2):e23612. doi: 10.2196/23612.

114. Thom D, Ortega J, Felix R, editors. NutrIA: An Out-of-the-Standard Nutritional Recommendation Mobile Application Powered by Artificial Intelligence. 2024 IEEE 4th International Conference on Advanced Learning Technologies on Education & Research (ICALTER); 2024 10-12 Dec. 2024.

115. Kumar H, Yoo S, Bernuy AZ, Shi J, Luo H, Williams JJ, et al. Large Language Model Agents for Improving Engagement with Behavior Change Interventions: Application to Digital Mindfulness. Proc ACM Hum-Comput Interact. 2025;9(7):Article CSCW438. doi: 10.1145/3757619.

116. Figueroa CA, Torkamaan H, Bhattacharjee A, Hauptmann H, Guan KW, Sedrakyan G. Designing Health Recommender Systems to Promote Health Equity: A Socioecological Perspective. J Med Internet Res. 2025 2025/1/30;27:e60138. doi: 10.2196/60138.

117. Raggioli L, Ciccarelli F, Rossi S, Rossi A, editors. A Robotic Assistant for Personalised Diet Recommendation. 2025 34th IEEE International Conference on Robot and Human Interactive Communication (RO-MAN); 2025 25-29 Aug. 2025.

118. González-Daza BA, Feijóo-García MA, editors. Fynex: Work in Progress on a Web-based Approach That Implements a Hybrid Recommendation System for Preventing and Treating Diseases based on Eating Disorders. CHIRA; 2022.

119. Maharjan R, Mendu S, Mariani M, Abdullah S, Hansen JP. Exploring user engagement with real-time verbal feedback from an exoskeleton-based virtual exercise coach. DIGITAL HEALTH. 2024 2024/09/01;10:20552076241302652. doi: 10.1177/20552076241302652.

120. Hoang YN, Chen S-H, Chang C-C, Lin AW, Nguyen TH, Hung LX, et al. Trust Predicts Actual Use of AI Chatbot as a Virtual Nutrition Assistant Among Dietetic Students in Taiwan: A Path Analysis. Journal of Human Nutrition and Dietetics. 2025 2025/12/01;38(6):e70156. doi: https://doi.org/10.1111/jhn.70156.

121. Mai V, Bauer A, Deggelmann C, Richert A, editors. Acceptance and User Needs of Coaching Chatbots: An Empirical Analysis of a StudiCoachBot’s Conversation Histories. HCI International 2023 – Late Breaking Papers; 2023 2023//; Cham: Springer Nature Switzerland.

122. Li J, Maharjan B, Xie B, Tao C. A Personalized Voice-Based Diet Assistant for Caregivers of Alzheimer Disease and Related Dementias: System Development and Validation. J Med Internet Res. 2020 2020/9/21;22(9):e19897. doi: 10.2196/19897.

123. Martinho D, Crista V, Carneiro J, Matsui K, Corchado JM, Marreiros G. Effects of a Gamified Agent-Based System for Personalized Elderly Care: Pilot Usability Study. JMIR Serious Games. 2023 2023/11/23;11:e48063. doi: 10.2196/48063.

124. Lupetti ML, Hagens E, Maden WVD, Steegers-Theunissen R, Rousian M. Trustworthy Embodied Conversational Agents for Healthcare: A Design Exploration of Embodied Conversational Agents for the periconception period at Erasmus MC. Proceedings of the 5th International Conference on Conversational User Interfaces; Eindhoven, Netherlands: Association for Computing Machinery; 2023. p. Article 25.

125. Mauricio D, Flores-Cortegana CM, Shuan-Arias AJ, Castañeda P, Rojas-Mezarina L, Castillo-Sequera JL. Sedentary: A Healthy Lifestyle App for Home Office Workers. International Journal of Interactive Mobile Technologies (iJIM). 2025 04/25;19(08):pp. 188-209. doi: 10.3991/ijim.v19i08.49147.

126. Meywirth S, Janson A, Söllner M, editors. Personalized coaching for lifestyle behavior change through large language models: A qualitative study. Hawaii International Conference on System Sciences (HICSS); 2025.

127. Mauriello ML, Tantivasadakarn N, Mora-Mendoza MA, Lincoln ET, Hon G, Nowruzi P, et al. A Suite of Mobile Conversational Agents for Daily Stress Management (Popbots): Mixed Methods Exploratory Study. JMIR Form Res. 2021 2021/9/14;5(9):e25294. doi: 10.2196/25294.

128. Meywirth S, Janson A, Söllner M, editors. Designing for Trust: Integrating Self-referencing in Large Language Model-Based Health Coaching. Local Solutions for Global Challenges; 2025 2025//; Cham: Springer Nature Switzerland.

129. Garcia MB, Mangaba JB, Tanchoco CC, editors. Acceptability, Usability, and Quality of a Personalized Daily Meal Plan Recommender System: The Case of Virtual Dietitian. 2021 IEEE 13th International Conference on Humanoid, Nanotechnology, Information Technology, Communication and Control, Environment, and Management (HNICEM); 2021 28-30 Nov. 2021.

130. Rasouli S, Ghafurian M, Dautenhahn K. Students’ Views on Intelligent Agents as Assistive Tools for Dealing with Stress and Anxiety in Social Situations. Proceedings of the 10th International Conference on Human-Agent Interaction; Christchurch, New Zealand: Association for Computing Machinery; 2022. p. 23–31.

131. Mishra V, Hong S, Kotz D. Exploring the Relationship Between Intrinsic Motivation and Receptivity to mHealth Interventions. Companion of the 2024 on ACM International Joint Conference on Pervasive and Ubiquitous Computing; Melbourne VIC, Australia: Association for Computing Machinery; 2024. p. 437–43.

132. Rasouli S, Ghafurian M, Nilsen ES, Dautenhahn K. University Students’ Opinions on Using Intelligent Agents to Cope with Stress and Anxiety in Social Situations. Computers in Human Behavior. 2024 2024/04/01/;153:108072. doi: https://doi.org/10.1016/j.chb.2023.108072.

133. Moore R, Al-Tamimi A-K, Freeman E. Investigating the Potential of a Conversational Agent (Phyllis) to Support Adolescent Health and Overcome Barriers to Physical Activity: Co-Design Study. JMIR Form Res. 2024 2024/1/31;8:e51571. doi: 10.2196/51571.

134. Rutjes H, Willemsen MC, IJsselsteijn WA. Beyond Behavior: The Coach's Perspective on Technology in Health Coaching. Proceedings of the 2019 CHI Conference on Human Factors in Computing Systems; Glasgow, Scotland Uk: Association for Computing Machinery; 2019. p. Paper 670.

135. Moreno-Blanco D, Solana-Sánchez J, Sánchez-González P, Jiménez-Hernando M, Cattaneo G, Roca A, et al. Intelligent Coaching Assistant for the Promotion of Healthy Habits in a Multidomain mHealth-Based Intervention for Brain Health. International Journal of Environmental Research and Public Health [Internet]. 2021; 18(20):[10774 p.].

136. Szymanski A, Wimer BL, Anuyah O, Eicher-Miller HA, Metoyer RA. Integrating Expertise in LLMs: Crafting a Customized Nutrition Assistant with Refined Template Instructions. Proceedings of the 2024 CHI Conference on Human Factors in Computing Systems; Honolulu, HI, USA: Association for Computing Machinery; 2024. p. Article 992.

137. Murali P, Shamekhi A, Parmar D, Bickmore TW, editors. Argumentation is More Important than Appearance for Designing Culturally Tailored Virtual Agents. AAMAS; 2020.

138. Torkamaan H, Ziegler J. Rating-based Preference Elicitation for Recommendation of Stress Intervention. Proceedings of the 27th ACM Conference on User Modeling, Adaptation and Personalization; Larnaca, Cyprus: Association for Computing Machinery; 2019. p. 46–50.

139. Nelekar S, Abdulrahman A, Gupta M, Richards D. Effectiveness of embodied conversational agents for managing academic stress at an Indian University (ARU) during COVID-19. British Journal of Educational Technology. 2022 2022/05/01;53(3):491-511. doi: https://doi.org/10.1111/bjet.13174.

140. Tseng Y-C, Chen S, Mah K-H, Chen Y-C, editors. Designing an AI Chatbot for Team-Based Diabetes Care: An Iterative Human-in-the-Loop Approach. Cross-Cultural Design; 2025 2025//; Cham: Springer Nature Switzerland.

141. Oc Y, Plangger K. GIST do it! How motivational mechanisms help wearable users develop healthy habits. Computers in Human Behavior. 2022 2022/03/01/;128:107089. doi: https://doi.org/10.1016/j.chb.2021.107089.

142. Arun C, Chaudhari D, Jaleel SA, Kannan SR, Selvakumarasamy S, Karthick S, editors. DiabeDoc: Intelligent Chatbot Prescriptions Based on Medical History and Lifestyle. 2025 2nd International Conference on Computing and Data Science (ICCDS); 2025 25-26 July 2025.

143. Ono S, Yotsuya Y, Takahashi N, Sakamoto T, Kato T, editors. Physical-exercise recommendation system based on individual daily schedule. 2022 Joint 12th International Conference on Soft Computing and Intelligent Systems and 23rd International Symposium on Advanced Intelligent Systems (SCIS&ISIS); 2022 29 Nov.-2 Dec. 2022.

144. Yan J, Ren L, Zhang Q, Zhang D. Research on Interaction Design of Personalized Intelligent Exercise System for Seniors in China. Proceedings of the 4th International Conference on Biomedical and Intelligent Systems: Association for Computing Machinery; 2025. p. 266–72.

145. Park J, Chung SY, Park JH. Real-Time Exercise Feedback through a Convolutional Neural Network: A Machine Learning-Based Motion-Detecting Mobile Exercise Coaching Application. Yonsei Med J. 2022 Jan;63(Suppl):S34-s42. PMID: 35040604. doi: 10.3349/ymj.2022.63.S34.

146. Diaz V, Carrasco F, Solis R, editors. Mobile Platform for Personalized Nutritional Recommendations Using a Machine-Learning Cloud Service for Patients with Chronic Diseases. 2025 13th International Conference in Software Engineering Research and Innovation (CONISOFT); 2025 27-31 Oct. 2025.

147. Patra E, Kokkinopoulou A, Wilson-Barnes S, Hart K, Gymnopoulos LP, Tsatsou D, et al. Personal Goals, User Engagement, and Meal Adherence within a Personalised AI-Based Mobile Application for Nutrition and Physical Activity. Life [Internet]. 2024; 14(10):[1238 p.].

148. Aharony N, Krakovski M, Edan Y. A Transparency-Based Action Model Implemented in a Robotic Physical Trainer for Improved HRI. J Hum-Robot Interact. 2024;14(1):Article 15. doi: 10.1145/3700598.

149. Paul SC, Bartmann N, Clark JL. Customizability in Conversational Agents and Their Impact on Health Engagement (Stage 2). Human Behavior and Emerging Technologies. 2024 2024/01/01;2024(1):5015913. doi: https://doi.org/10.1155/2024/5015913.

150. Dindorf C, Dully J, Bartaguiz E, Menges T, Reidick C, Seibert J-N, et al. Characteristics and perceived suitability of artificial intelligence-driven sports coaches: a pilot study on psychological and perceptual factors. Frontiers in Sports and Active Living. 2025 2025-May-12;Volume 7 - 2025. doi: 10.3389/fspor.2025.1548980.

151. Piao M, Kim J, Ryu H, Lee H. Development and usability evaluation of a healthy lifestyle coaching chatbot using a habit formation model. Healthcare Informatics Research. 2020;26(4):255-64. doi: 10.4258/hir.2020.26.4.255.

152. Herter E, Horstmann AC. Judged by a Chatbot? An Empirical Investigation of the Impact of Expectancy Violations on Users' Trust in AI-based Chatbots. Proceedings of the 25th ACM International Conference on Intelligent Virtual Agents: Association for Computing Machinery; 2025. p. Article 21.

153. Putta M, Sandbulte J, editors. Examining Personalized Explainable Recommendations that Support College Students on Stress Management. IoT Technologies and Wearables for HealthCare; 2025 2025//; Cham: Springer Nature Switzerland.

154. Hoffman V, Flom M, Mariano TY, Chiauzzi E, Williams A, Kirvin-Quamme A, et al. User Engagement Clusters of an 8-Week Digital Mental Health Intervention Guided by a Relational Agent (Woebot): Exploratory Study. J Med Internet Res. 2023 2023/10/13;25:e47198. doi: 10.2196/47198.

155. Rabbi M, Aung MSH, Gay G, Reid MC, Choudhury T. Feasibility and Acceptability of Mobile Phone–Based Auto-Personalized Physical Activity Recommendations for Chronic Pain Self-Management: Pilot Study on Adults. J Med Internet Res. 2018 2018/10/26;20(10):e10147. doi: 10.2196/10147.

156. Kannampallil T, Ronneberg CR, Wittels NE, Kumar V, Lv N, Smyth JM, et al. Design and Formative Evaluation of a Virtual Voice-Based Coach for Problem-solving Treatment: Observational Study. JMIR Form Res. 2022 2022/8/12;6(8):e38092. doi: 10.2196/38092.

157. Rabbi M, Aung MH, Zhang M, Choudhury T. MyBehavior: automatic personalized health feedback from user behaviors and preferences using smartphones. Proceedings of the 2015 ACM International Joint Conference on Pervasive and Ubiquitous Computing; Osaka, Japan: Association for Computing Machinery; 2015. p. 707–18.

158. Difini GM, Martins MG, Barbosa JLV. A Movement Analysis Application using Human Pose Estimation and Action Correction. Proceedings of the Brazilian Symposium on Multimedia and the Web; Curitiba, Brazil: Association for Computing Machinery; 2022. p. 359–67.

159. Radha M, Willemsen MC, Boerhof M, IJsselsteijn WA. Lifestyle Recommendations for Hypertension through Rasch-based Feasibility Modeling. Proceedings of the 2016 Conference on User Modeling Adaptation and Personalization; Halifax, Nova Scotia, Canada: Association for Computing Machinery; 2016. p. 239–47.

160. Ritschel H, Seiderer A, Janowski K, Aslan I, André E. Drink-O-Mender: An Adaptive Robotic Drink Adviser. Proceedings of the 3rd International Workshop on Multisensory Approaches to Human-Food Interaction; Boulder, CO, USA: Association for Computing Machinery; 2018. p. Article 3.

161. Rice A, Klęczek K, Alimardani M, editors. The Effectiveness of Social Robots in Stress Management Interventions for University Students. Social Robotics; 2024 2024//; Singapore: Springer Nature Singapore.

162. Spierling Bagsic SR, Savin KL, Soriano EC, San Diego ERN, Orendain N, Clark T, et al. Process evaluation of Dulce Digital-Me: an adaptive mobile health (mHealth) intervention for underserved Hispanics with diabetes. Translational Behavioral Medicine. 2023;13(9):635-44. doi: 10.1093/tbm/ibad020.

163. Robinson NL, Connolly J, Hides L, Kavanagh DJ, editors. A Social Robot to Deliver an 8-Week Intervention for Diabetes Management: Initial Test of Feasibility in a Hospital Clinic. Social Robotics; 2020 2020//; Cham: Springer International Publishing.

164. Sun X, Liu Y, Bosch JA, Li Z. Interface Matters: Exploring Human Trust in Health Information from Large Language Models via Text, Speech, and Embodiment. Proc ACM Hum-Comput Interact. 2025;9(2):Article CSCW116. doi: 10.1145/3711014.

165. Suwan R, Temdee P, Prasad R. Context-aware Based Personalized Recommendation on Mobile for Monitoring Excessive Sugar Consumption of Thai Adolescents. Journal of Mobile Multimedia. 2022 07/18;18(06):1879-912. doi: 10.13052/jmm1550-4646.18618.

166. Wu PF, Summers C, Panesar A, Kaura A, Zhang L. AI Hesitancy and Acceptability—Perceptions of AI Chatbots for Chronic Health Management and Long COVID Support: Survey Study. JMIR Hum Factors. 2024 2024/7/23;11:e51086. doi: 10.2196/51086.

167. Shamekhi A, Bickmore T. Breathe Deep: A Breath-Sensitive Interactive Meditation Coach. Proceedings of the 12th EAI International Conference on Pervasive Computing Technologies for Healthcare; New York, NY, USA: Association for Computing Machinery; 2018. p. 108–17.

168. Yang T-t, Zheng H-x, Cao S, Jing M-l, Hu J, Zuo Y, et al. Harnessing an Artificial Intelligence–Based Large Language Model With Personal Health Record Capability for Personalized Information Support in Postsurgery Myocardial Infarction: Descriptive Qualitative Study. J Med Internet Res. 2025 2025/4/30;27:e68762. doi: 10.2196/68762.

169. Shen Z, Wu Y. Investigation of Practical Use of Humanoid Robots in Elderly Care Centres. Proceedings of the Fourth International Conference on Human Agent Interaction; Biopolis, Singapore: Association for Computing Machinery; 2016. p. 63–6.

170. Abbas A, Wohn C, Jagtap A, Rho EH, Kim Y-H, Lee SW. "Having Lunch Now": Understanding How Users Engage with a Proactive Agent for Daily Planning and Self-Reflection. Proceedings of the 2026 CHI Conference on Human Factors in Computing Systems: Association for Computing Machinery; 2026. p. Article 1332.

171. Starke A, El Majjodi A, Trattner C, Brusilovsky P, Gemmis M, Felfernig A, editors. Boosting Health? Examining the Role of Nutrition Labels and Preference Elicitation Methods in Food Recommendation. IntRS@ RecSys; 2022.

172. McCarren L, Eriksson U, Mengual LO, Kuoppamäki S. Exploring the Design of a LLM-Based AI Assistant for Mindfulness Practice With Older Adults. Proceedings of the 2026 CHI Conference on Human Factors in Computing Systems: Association for Computing Machinery; 2026. p. Article 253.

173. Starke AD, Musto C, Rapp A, Semeraro G, Trattner C. “Tell Me Why”: using natural language justifications in a recipe recommender system to support healthier food choices. User Modeling and User-Adapted Interaction. 2024 2024/04/01;34(2):407-40. doi: 10.1007/s11257-023-09377-8.

174. Wu M, Jiang Z, Fan Y, Feng R, Dharmavaram S, Polowitz M, et al. MindfulAgents: Personalizing Mindfulness Meditation via an Expert-Aligned Multi-Agent System. Proceedings of the 2026 CHI Conference on Human Factors in Computing Systems: Association for Computing Machinery; 2026. p. Article 1025.

175. Stephens TN, Joerin A, Rauws M, Werk LN. Feasibility of pediatric obesity and prediabetes treatment support through Tess, the AI behavioral coaching chatbot. Translational Behavioral Medicine. 2019;9(3):440-7. doi: 10.1093/tbm/ibz043.

176. Zhang X, Hu M, Xue B, Purwanto E, Selig TJ, Yonto D. SmartWalkCoach: An AI Companion for End-to-End Walking Guidance, Motivation, and Reflection. Proceedings of the 31st International Conference on Intelligent User Interfaces: Association for Computing Machinery; 2026. p. 1004–18.

177. Sze WT, Waki K, Enomoto S, Nagata Y, Nangaku M, Yamauchi T, et al. StepAdd: A personalized mHealth intervention based on social cognitive theory to increase physical activity among type 2 diabetes patients. Journal of Biomedical Informatics. 2023 2023/09/01/;145:104481. doi: https://doi.org/10.1016/j.jbi.2023.104481.

178. Goulart C, Lopes Alves M, D’Angelo Medeiros F, Fernando Borges R, Lima Rodrigues G, Cristina De Araújo Alves C, et al. Utilizing artificial intelligence for exercise prescription and heart rate monitoring in cardiorespiratory rehabilitation. European Respiratory Journal.64(suppl 68):PA602. doi: 10.1183/13993003.congress-2024.PA602.

179. Thongyoo P, Anantapanya P, Jamsri P, Chotipant S, editors. A Personalized Food Recommendation Chatbot System for Diabetes Patients. Cooperative Design, Visualization, and Engineering; 2020 2020//; Cham: Springer International Publishing.

180. Dinç H, Argan M, Çevik H, Gürbüz B, Koçak F, Tokay Argan M. Investigating the Adoption Intention of ChatGPT for Exercise Prescription: An Integrated Model of the Extended Technology Acceptance Model (TAM) and The Theory of Planned Behavior (TPB). Available at SSRN 5477206.

181. Lara MMdJT, Cruz AJA, Rey YV, editors. Preliminary Results of Intelligent Model for Food Recommendation Consumption in Adults. 2023 18th Iberian Conference on Information Systems and Technologies (CISTI); 2023 20-23 June 2023.

182. Murphy K, Davis C, Curtis R, Maher C. Delivery of a 3-month Mediterranean diet and physical activity lifestyle intervention via artificial-intelligence chatbot, can achieve behaviour change: MedLiPal pilot-study. Proceedings of the Nutrition Society. 2020;79(OCE2):E146. doi: 10.1017/S0029665120000944.

183. To QG, Green C, Vandelanotte C. Feasibility, Usability, and Effectiveness of a Machine Learning–Based Physical Activity Chatbot: Quasi-Experimental Study. JMIR Mhealth Uhealth. 2021 2021/11/26;9(11):e28577. doi: 10.2196/28577.

184. Mathews S, Jeba SVA. Everfitme: AI-powered fitness application. AIP Conference Proceedings. 2025;3298(1):020045. doi: 10.1063/5.0279844.

185. Torkamaan H, Ziegler J. Recommendations as Challenges: Estimating Required Effort and User Ability for Health Behavior Change Recommendations. Proceedings of the 27th International Conference on Intelligent User Interfaces; Helsinki, Finland: Association for Computing Machinery; 2022. p. 106–19.

186. Hart K, Wilson-Barnes S, Lanham-New SA, Gymnopoulos L, Dimitropoulos K, Stefanidis K, et al. Can a mobile application deliver appropriate, acceptable and usable personalised nutrition advice to UK adults. Proceedings of the Nutrition Society. 2023;82(OCE5):E314. doi: 10.1017/S0029665123004135.

187. Triantafyllidis A, Alexiadis A, Elmas D, Gerovasilis G, Votis K, Tzovaras D. A social robot-based platform for health behavior change toward prevention of childhood obesity. Universal Access in the Information Society. 2023 2023/11/01;22(4):1405-15. doi: 10.1007/s10209-022-00922-7.

188. Ng W, Tay P, Tan J, Chan Y, Tong C. NutBot–Ask Anne: Evaluation on nutrition chatbot to support patients with malnutrition. Clinical Nutrition ESPEN. 2025;69:1115-6.

189. Vandeputte J, Herold P, Kuslii M, Viappiani P, Muller L, Martin C, et al. Principles and Validations of an Artificial Intelligence-Based Recommender System Suggesting Acceptable Food Changes. The Journal of Nutrition. 2023 2023/02/01/;153(2):598-604. doi: https://doi.org/10.1016/j.tjnut.2022.12.022.

190. Trivedi R, Shaw T, Chow CK, Laranjo L. Conversational artificial intelligence intervention to support patients with atrial fibrillation: process evaluation of a randomised controlled trial. European Heart Journal. 2023;44(Supplement_2):ehad655.2995. doi: 10.1093/eurheartj/ehad655.2995.

191. Vardhan M, Hegde N, Merugu S, Prabhat S, Nathani D, Seneviratne M, et al. Walking with PACE - Personalized and Automated Coaching Engine. Proceedings of the 30th ACM Conference on User Modeling, Adaptation and Personalization; Barcelona, Spain: Association for Computing Machinery; 2022. p. 57–68.

192. Zhao Y, Qian C, Guo W, Zhu W, Zheng P. Design and evaluation of a personalized mHealth intervention system based on machine learning to promote smoking cessation in China. Tob Induc Dis. 2025 2025;23(1).

193. Vemuri A, Heintzelman M, Waad A, Mauriello ML, Decker K, Dominick G. Towards Dynamic Action Planning with user preferences in Automated Health Coaching. Smart Health. 2023 2023/06/01/;28:100389. doi: https://doi.org/10.1016/j.smhl.2023.100389.

194. Leveraging Digital Health Innovations in Obesity Management. Endocrine practice : official journal of the American College of Endocrinology and the American Association of Clinical Endocrinologists. 2025;31:S214. doi: 10.1016/j.eprac.2025.05.630.

195. V K, Thiyagu TM, Taurshia A, Nagarajan B, J A, I JFJ, editors. FDM: NutriGuide - A Cross-Platform Flutter Application for Managing and Educating on Nutritional Deficiencies. 2024 Second International Conference on Intelligent Cyber Physical Systems and Internet of Things (ICoICI); 2024 28-30 Aug. 2024.

**Detailed system characteristics and feature coding underlying Figure 2.**

| **Feature dimension** | **Category** | **n (%)** | **Brief description** |
| --- | --- | --- | --- |
| System format | LLM-centered conversational system | 12 (57.1%) | Chatbot, voice, or agentic conversational systems using LLMs such as GPT-series models, Claude, or Gemini [1-12]. |
|  | Hybrid AI system | 6 (28.6%) | Systems combining LLM generation with other components, such as wearables, bandits, ML classifiers, IoT sensing, rule validation, or agent-persona architecture [13-18]. |
|  | Learning/optimization-based recommender | 3 (14.3%) | Non-LLM recommender systems using reinforcement learning or multi-objective optimization to construct personalized recommendations [19-21]. |
| Core AI method | LLM prompting/generation | 12 (57.1%) | LLM-based generation using prompt engineering, conversational workflows, or agentic generation [1-5, 7, 8, 10-12, 14, 17]. |
|  | KG/RAG-grounded LLM pipeline | 3 (14.3%) | Systems employing KG retrieval and RAG to ground LLM outputs [6, 9, 13]. |
|  | ML-supported decision-making + LLM generation | 2 (9.5%) | ML models or adaptive decision components used before or alongside LLM-generated recommendations [15, 16]. |
|  | Constrained AIGC + rule validation | 1 (4.8%) | Generative content constrained or checked using expert rules, structured knowledge, filters, or fallback templates [18]. |
|  | Learning/optimization-based algorithms | 3 (14.3%) | Reinforcement learning or evolutionary optimization used to generate personalized recommendation sequences or plans [19-21]. |
| Data input | Demographics & basic profile | 12 (57.1%) | Age, gender, location, language, or other basic user profile information [1-5, 10, 12, 17-21] |
|  | Health/physiology & biometrics | 14 (66.7%) | Health status, symptoms, clinical indicators, physiological data, or biometric measures [6, 9-21]. |
|  | Behaviors & habits/history | 16 (76.2%) | Historical behaviors, routines, lifestyle habits, prior activity, or past recommendation history [1-4, 6, 9-11, 13, 15-21]. |
|  | Goals & intentions | 12 (57.1%) | User-defined goals, intentions, health targets, or desired outcomes [1, 2, 4-7, 9, 10, 12, 17, 19, 21]. |
|  | Preferences & constraints | 15 (71.4%) | Dietary preferences, exercise preferences, restrictions, budget, time, allergies, or other constraints [1, 2, 4-7, 9, 10, 12, 13, 17-21]. |
|  | Mood/psychological status | 2 (9.5%) | Emotional, stress-related, or psychological state information [4, 14]. |
|  | Context/situation | 8 (38.1%) | Real-time or situational context such as time, weather, location, or available resources [3, 4, 9, 10, 14, 15, 17, 18]. |
|  | Feedback & adherence signals | 10 (47.6%) | User feedback, adherence, uptake, response, or follow-up signals used for refinement [5, 9-11, 15-20]. |
|  | Interaction content & history | 11 (52.4%) | Conversation history, user messages, prior interactions, or interaction summaries [1-3, 5, 8-11, 14, 15, 17]. |
| Knowledge source | System prompts / LLM-internal knowledge | 11 (52.4%) | Systems relying mainly on prompt-encoded principles, model knowledge, or internally generated reasoning [1-5, 7, 8, 12, 14-16]. |
|  | External guidelines / databases / knowledge bases | 10 (47.6%) | Systems grounded in external sources such as guidelines, recipe databases, exercise libraries, knowledge graphs, or structured health resources [6, 9-11, 13, 17-21]. |
| Delivery channel/interface | Application-based interface | 13 (61.9%) | Mobile, web, messaging-app, dashboard, or voice-enabled application interfaces [3-5, 9, 11, 12, 15-21]. |
|  | General-purpose AI platform | 5 (23.8%) | Systems implemented directly through platforms such as ChatGPT or similar general-purpose conversational AI interfaces [1, 2, 7, 8, 14] |
|  | Physical embodiment | 1 (4.8%) | Robot or device-based delivery [13]. |
|  | Not explicitly specified | 2 (9.5%) | Delivery channel not clearly reported [6, 10]. |

1. Ataguba G, Orji R. Exploring Large Language Models for Personalized Recipe Generation and Weight-Loss Management. ACM Trans Comput Healthcare. 2025;6(2):Article 22. doi: 10.1145/3712709.

2. Ataguba G, Oyebode O, Orji F, Henry KC, Orji R, editors. Persuasion and Behavior Change in ChatGPT-Based Dietary Management. 2025 IEEE Conference on Serious Games and Applications for Health (SeGAH); 2025.

3. Larbi D, Løvas SE, Årsand E, Denecke K, Gabarron E, Henriksen A. Designing and testing a physical activity app with a Chatgpt-based Chatbot. Intelligent Health Systems–From Technology to Data and Knowledge: IOS Press; 2025. p. 1155-9.

4. Liang M, Luo Y, editors. Exploring Multi-LLM Collaboration to Power Conversational Recommender System: A Case Study of Dietary Recommendation. Proceedings of the 7th ACM Conference on Conversational User Interfaces; 2025.

5. Liang M, Wang J, Luo Y, editors. SmartEats: Investigating the Effects of Customizable Conversational Agent in Dietary Recommendations. Proceedings of the 7th ACM Conference on Conversational User Interfaces; 2025.

6. Liu L, Zhang S, Chen M, Shi X, Li G, editors. KG-DietNet: A Personalized Dietary Recommendation System Based on Knowledge Graphs and Large Language Models. 2025 IEEE 12th Joint International Information Technology and Artificial Intelligence Conference (ITAIC); 2025.

7. Meywirth S, editor. Designing a Large Language Model-Based Coaching Intervention for Lifestyle Behavior Change. Design Science Research for a Resilient Future; 2024; Cham: Springer Nature Switzerland.

8. Phan TA, Nguyen TH-T, Nguyen C. “Hey AI, What Should I Eat?” Navigating Skepticism and Trust in AI-Powered Meal Recommendations Through Personalized Persuasion. International Journal of Human–Computer Interaction. 2025 2025/11/03:1-13. doi: 10.1080/10447318.2025.2575098.

9. Gao F, Zhao X, Xia D, Zhou Z, Yang R, Lu J, et al. HealthGenie: A Knowledge-Driven LLM Framework for Tailored Dietary Guidance. Proceedings of the 34th ACM International Conference on Information and Knowledge Management; Seoul, Republic of Korea: Association for Computing Machinery; 2025. p. 6639–43.

10. Goh HL, Sancenon V, Chu BMX, Koh GCH, Koh L, Teo D, et al. Personalised health plan development using agentic AI in Singapore’s national preventive care programme: a pilot study. npj Digital Medicine. 2026 2026/03/09;9(1):332. doi: 10.1038/s41746-026-02514-8.

11. Liu X, Liu J. Assessing the Feasibility, Usability, Acceptability, and Efficacy of an AI Chatbot for Sleep Promotion: Quasi-Experimental Study. JMIR Form Res. 2026 2026/2/3;10:e84023. doi: 10.2196/84023.

12. Tyagi V, Sharma V, Alam M, Baliyaan S, editors. Smart Wellness through Conversational AI: Building FitVore with Gemini for Personalized Health Coaching. 2025 2nd Global AI Summit - International Conference on Artificial Intelligence and Emerging Technology (AI Summit); 2025 19-21 Nov. 2025.

13. Jahn ET, Nunes SS, Bevilacqua R, Stara V, Margaritini A, Lehmann J, et al. Different Uses of LLM based Digital Health Coaching Devices for Community-Dwelling Older Adults in Europe and Japan - Lessons Learned from an international Research Project. Proceedings of the 12th International Conference on Communities & Technologies: Association for Computing Machinery; 2025. p. 12–27.

14. Neupane S, Dongre P, Gracanin D, Kumar S. Wearable Meets LLM for Stress Management: A Duoethnographic Study Integrating Wearable-Triggered Stressors and LLM Chatbots for Personalized Interventions. Proceedings of the Extended Abstracts of the CHI Conference on Human Factors in Computing Systems: Association for Computing Machinery; 2025. p. Article 588.

15. Wang X, Griffith J, Adler DA, Castillo J, Choudhury T, Wang F. Exploring Personalized Health Support through Data-Driven, Theory-Guided LLMs: A Case Study in Sleep Health. Proceedings of the 2025 CHI Conference on Human Factors in Computing Systems: Association for Computing Machinery; 2025. p. Article 507.

16. C. V R, D IH, J. G LK, Sujihelen L, Srividhya E, Jayanthi S, editors. AI-Powered Parkinson's Wellness Assistant Featuring Physiotherapy Guidance, Diet Management, and Cognitive Exercises. 2026 Contemporary Computing Innovations Conference (CCIC); 2026 6-7 Feb. 2026.

17. Gao B, Zeng Z, Yu Y, Werry IP, Chan CL, Chen M, et al. "It Seems to Understand My Heart": An Empirical Study of Persona-Driven Persuasive AI Agent for Aging-in-Place in Singapore. Proceedings of the 2026 CHI Conference on Human Factors in Computing Systems: Association for Computing Machinery; 2026. p. Article 992.

18. Sheng Y, Li M, Yang L. AIGC-Driven Personalized Traditional Chinese Medicine Dietary Therapy Interactive System: Design and Implementation. Proceedings of the 2025 2nd International Conference on Artificial Intelligence, Digital Media Technology and Interaction Design: Association for Computing Machinery; 2026. p. 160–6.

19. Tragos EZ, O'Reilly-Morgan D, Geraci J, Shi B, Smyth B, Doherty C, et al., editors. Keeping People Active and Healthy at Home Using a Reinforcement Learning-based Fitness Recommendation Framework. IJCAI; 2023.

20. Doherty C, Lambe R, O’Grady B, O’Reilly-Morgan D, Smyth B, Lawlor A, et al. An Evaluation of the Effect of App-Based Exercise Prescription Using Reinforcement Learning on Satisfaction and Exercise Intensity: Randomized Crossover Trial. JMIR Mhealth Uhealth. 2024 2024/11/26;12:e49443. doi: 10.2196/49443.

21. Alcaraz-Herrera H, Cartlidge J, Toumpakari Z, Western M, Palomares I. EvoRecSys: Evolutionary framework for health and well-being recommender systems. User Modeling and User-Adapted Interaction. 2022 2022/11/01;32(5):883-921. doi: 10.1007/s11257-021-09318-3.
